# Changes in gene regulation are associated with the evolution of resistance to a novel parasite

**DOI:** 10.3389/fimmu.2026.1697157

**Published:** 2026-02-26

**Authors:** Lauren E. Fuess, Amanda K. Hund, Mariah L. Kenney, Meghan F. Maciejewski, Joseph M. Marini, Daniel I. Bolnick

**Affiliations:** 1 Department of Biology, Texas State University, San Marcos, TX, United States; 2 Department of Ecology and Evolutionary Biology, University of Connecticut, Storrs, CT, United States; 3 Department of Biology, Carleton College, Northfield, MN, United States; 4 School of Integrative Biology, University of Illinois at Urbana-Champaign, Urbana, IL, United States

**Keywords:** ecoimmunology, evolutionary immunology, fibrosis, host parasite, stickleback, transcriptomics

## Abstract

**Introduction:**

Host–parasite interactions are ubiquitous and are important drivers of host diversification and evolution. In particular, host immune systems are frequent targets of parasite-driven selection. The resulting rapid evolution of immune genes is usually framed as an ongoing ‘arms race’ between a co-evolving pair of host and parasite species. However, immune evolution may often be driven by the acquisition of a new and unfamiliar parasite. For instance, when marine populations of threespine stickleback (*Gasterosteus aculeatus*) colonized freshwater lakes approximately 12,000 years ago, they encountered the freshwater-restricted cestode *Schistocephalus solidus* and evolved resistance.

**Methods:**

We compared the transcriptomic responses of lab-reared sticklebacks from three populations of stickleback with varying cestode susceptibilities when exposed to several immune stimuli (alum, cestode protein, or a control injection).

**Results:**

The resulting changes in expression reveal strong evidence of shared and population-specific responses during the evolution of defense against a new parasite. Our investigation highlights the roles of several key immunological processes in underlying a general physiological response to tissue damage (fibrosis) and the importance of regulating fibrosis as a necessary step for its co-option into defense against *S. solidus* tapeworms. Furthermore, we highlighted changes in the expression of fibrosis-associated genes, which facilitate faster and more targeted deployment of this defense mechanism against parasites. Fish from the most fibrosis-prone population exhibited constitutively higher expression of fibrosis-associated genes and stronger downregulation of these genes after an initial stimulus from injected cestode proteins.

**Conclusion:**

Our results provide strong evidence that changes in gene regulation and increased negative feedback to mitigate immunopathology are essential steps in the evolutionary co-option of an existing pathway to defend against a new parasite infection.

## Introduction

The vertebrate immune system comprises many evolutionarily ancient genes and biological processes (1). For instance, the NF-κB signaling pathway, which is central to innate immunity, likely originated in the common ancestor of metazoans or even earlier, and many of its functions are highly conserved across animals (2). Juxtaposed against this evolutionary conservatism, parasites can impose strong natural selection on host immune systems, even over short time scales (3, 4). Selective forces induced by host–parasite interactions were first described in Van Valen’s Red Queen’s Race theory (5) and have been widely studied (6–10). Parasitism has been shown to drive substantial immune diversification in animals, and some immune genes are among the fastest-evolving genes (11, 12). How can we resolve this apparent contradiction between the evolutionary conservatism and rapid evolution of animals’ immune systems? One possible explanation is that contemporary microevolution might rely on minor modifications or co-option of existing genes and pathways (13–15), an example of the ‘evolutionary tinkering’ proposed by Jacob in 1977 (16). Therefore, an important question in evolutionary immunology is whether animal adaptation to parasites mostly involves adjustments to existing immune pathways, loss of old pathways, or co-option of existing pathways for new purposes. This question is particularly relevant to situations in which a host population encounters a novel parasite species and subsequently evolves novel defenses (as opposed to the long-term coevolution that is the focus of the Red Queen theory).

However, the evolution of immune defenses against novel parasites is not simply a gain of function. Animals must also suppress their defense when it is not required and de-escalate after a successful response. Parasite defenses can be costly in a multitude of ways; excessive immune responses can cause host damage [i.e., “immunopathology,” (17–19)], and immunity is energetically costly, drawing resources from other beneficial processes [e.g., reproduction and growth rate; (20–22)]. Natural selection does not typically favor ever-greater resistance but rather an optimal balance between immune costs and benefits (23). Because of this balancing selection, adaptation to new parasites could drive either the evolution of up- or downregulation of immune genes in a tightly coordinated series of events and might promote either the gain or loss of genes or pathways. Here we report the results of a study that evaluated the extent to which adaptation to a new parasite involves gain versus loss, up- versus downregulation, and co-option or novelty of immune gene expression and pathways in a model host–parasite system.

Recent studies have identified an example of an immunological gain of function during adaptation to a new parasite. The three-spined stickleback (*Gasterosteus aculeatus*) is host to a specialist cestode, *Schistocephalus solidus*. Sticklebacks are typically marine or anadromous (migrating from the ocean into brackish estuaries to breed). During Pleistocene deglaciation (approximately 12,000 years ago), marine sticklebacks established permanent land-locked populations in newly available lakes throughout the coastal north-temperate regions (24). This unique evolutionary history, with extant ancestral marine sticklebacks and thousands of replicated freshwater populations, has been widely used to study the repeatability of evolution and the genetic basis of adaptation (25–27). Most notably, heavily armored marine fish almost always evolve reduced armor plating after establishing populations in freshwater (28, 29). However, armor defense against predators was not the only key adaptation to freshwater. When marine sticklebacks colonize freshwater, they encounter the cestode *S. solidus*, which is exclusive to freshwater because its eggs do not hatch in brackish water (30, 31). Because they do not encounter *S. solidus* in marine habitats, marine sticklebacks that colonize freshwater would have been highly susceptible to this parasite (31) and become heavily infected (32). Permanent freshwater populations subsequently evolved immune adaptations to resist *S. solidus* infections (31). For example, in 1968, marine fish were experimentally introduced to a human-made quarry pond, rapidly infected, and evolved effective resistance [i.e., the ability to limit parasite growth or load (33)] over the subsequent 50 years, nearly eliminating the cestode by 2022 (32). This artificial population, like many natural lake populations, evolved a peritoneal-wide fibrosis response, which is induced by parasite exposure and contributes to suppressing parasite growth and viability (31). In other populations, fibrosis is actively suppressed after the initial infection, allowing the cestode to grow while avoiding pathological side effects of fibrosis. This has been defined as a tolerance strategy [i.e., the ability to minimize parasite pathogenicity without impacting growth/load (33)]. These differences in the fibrosis response are observable under laboratory conditions and are heritable (34). Importantly, marine sticklebacks, which are evolutionarily naive to *S. solidus*, lack a fibrosis response to cestodes (35, 36), although they possess the capacity for fibrosis when stimulated by artificial adjuvants. The fibrosis response and cestode resistance, thus, represent an example of an immune gain-of-function when ancestral marine fish colonized freshwater and adapted to resist *S. solidus*.

However, the general mechanisms underlying fibrosis and the evolutionary processes that led to its co-option into defense against a novel parasite in some stickleback populations remain poorly understood (35, 37). In particular, we do not currently know which gene expression cascades lead to the initiation of peritoneal fibrosis in sticklebacks. Nor do we know whether this regulatory cascade is a generic response to a pro-inflammatory stimulus (for instance) or whether it is specifically a response to *S. solidus*. Finally, we do not know whether the gene regulatory response that produces fibrosis is an ancient, conserved pathway shared by all stickleback populations or whether the expression response itself is evolving to differentially regulate fibrosis in diverging stickleback populations. That is, do marine sticklebacks have the same gene expression responses as their freshwater relatives? Do relatively resistant and susceptible freshwater populations exhibit the same regulatory changes when encountering *S. solidus*?

To answer such questions, we experimentally exposed lab-raised sticklebacks from three populations (ancestral marine, a relatively resistant lake population, and a relatively tolerant lake population) to different immune challenges (alum, an adjuvant; cestode protein extracts; and a saline control). A previous study reported the dynamics of the fibrosis response in this experiment, showing population differences in fibrosis responses (36). To expand on that prior result, here we present the temporal regulation of gene expression in response to these different immune challenges in the three populations. We evaluated the gene regulatory basis of fibrosis, response to cestode proteins, and population differences in these regulatory processes. We showed that population differences in fibrosis entail changes in the same overall gene regulatory pathways, including many evolutionarily ancient processes that were repurposed during adaptation to *S. solidus*. Nevertheless, there are also population-specific responses in some genes, which highlight the capacity for immune regulatory systems to exhibit substantial changes over microevolutionary time (approximately 10,000 generations).

## Methods

### Experimental design

The full details of the laboratory injection experiment can be found in Hund et al. (36). Briefly, *G. aculeatus* were collected in June of 2018 from populations on Vancouver Island, and reproductively mature adults were used for *in vitro* breeding to generate embryos. Fish were collected from three geographically isolated and genetically divergent populations (35). Sayward Estuary (SAY) is populated with anadromous sticklebacks that represent a proxy for the phenotypes and genotypes of cestode-susceptible ancestral marine fish that colonized freshwater lakes on Vancouver Island (38). Importantly, this ancestor-like population had very little natural exposure to *S. solidus*. In the laboratory, these fish are highly susceptible to *S. solidus* and demonstrate minimal fibrosis (34, 35). Roselle Lake (RSL) sticklebacks have a faster and stronger fibrosis response to infection (36) and consequently exhibit a lower infection prevalence in nature [7%–40%; (35, 39)]. The cestodes that establish in Roselle fish tend to be smaller or even encased in a granuloma that frequently leads to parasite death (Bolnick, *pers. obs.* & Hund, *unpublished data*). Because of this effective fibrosis response, we consider Roselle Lake fish to represent a resistant population, similar to the highly fibrotic Roberts Lake population studied previously (31). A second lake population, Gosling Lake (GOS), was chosen to represent a more tolerant low-fibrosis strategy. Since we began studying *S. solidus* infections in 2005, Gosling Lake sticklebacks have typically harbored a high prevalence of *S. solidus* (50%–80%), which grew rapidly to a large size without inducing discernible fibrosis. Genomic signatures of selection within the fibrosis QTL suggesting that this population has historically experienced selection favoring susceptible but tolerant genotypes that suppress the costly fibrosis response (35). We consider this population to be ‘tolerant,’ with the caveat that between 2012 and 2022, the population has been undergoing rapid evolution (and declining infection rates); therefore, at the time of sampling for this study, this population was likely polymorphic for fibrosis traits (40). Fish from each of the three populations were bred using standard *in vitro* fertilization methods to generate within-population crosses. The resulting fertilized eggs were transported to the laboratory for rearing (41). Fish were split across two aquarium rooms at the University of Connecticut, where they were reared for approximately 11 months prior to the start of the injection experiment (May 2019).

At the start of the experiment, fish from each population were given intraperitoneal injections of one of three inoculants: a control injection of 20 μL of 1× phosphate-buffered saline (PBS, control), a 20 μL injection of Alum (2% Alumax Phosphate, OZ Bioscience) diluted 1:1 in PBS, or a 20 μL injection of cestode protein homogenate diluted 1:1 in PBS. Alum is a common immune adjuvant that induces an innate immune response by recruiting leukocytes (42). Alum is widely used as an adjuvant in vaccination, although the mechanistic basis of this effect remains contested; it may serve as a deposit that slowly releases antigens or may help turn soluble antigens into particles for easier uptake by antigen-presenting cells. Alum also has antigen-independent effects, inducing inflammation by damaging cells to release damage-associated molecular patterns (DAMPs) (43), triggering CD8+ T cells (44), or inducing the endogenous uric acid ‘danger signal’ in dendritic cells (45). Regardless of the exact mechanism, evidence is emerging that alum induces fibrosis in a variety of model organisms, including liver fibrosis in mice (46) and in most species of fish (47), including both marine and freshwater sticklebacks (48). Alum injection serves as a positive control for fibrosis because all stickleback populations used here respond with increased fibrosis (36).

Cestode proteins were generated from *S. solidus* collected from a fourth location, Farwell Lake, which was collected in 2008, flash frozen, and stored at −80 until the time of experimentation. The use of cestode protein from a fourth population ensured that no population of fish was challenged with native parasites to which they might be locally adapted (or which might be locally adapted to them). Full details of the homogenate preparation can be found in Hund et al. (36). At the time of experimentation, the fish were lightly anesthetized using neutral-buffered MS-222 (50 mg/L–75 mg/L injected) and then injected with a randomly assigned treatment using sterile ultrafine syringes (BD 31G 8 mm). Injections were performed in the lower left peritoneal cavity using a shallow angle parallel to the body to prevent damage to the internal organs. Fish were also labeled with a small elastomer mark posterior to the neurocranium to allow for the identification of treatments (the fish were housed in common tanks). Following injection, the fish were allowed to recover from anesthesia before being returned to their original tanks. A full schematic of the experimental design is presented in **Supplementary Figure S1**.

Fish were euthanized using a lethal dose of neutral-buffered MS-222 for fibrosis measurement and collection of tissues (pronephros and peritoneal tissues) at four time points: 1-, 10-, 42-, and 90-days post-injection. Immediately following euthanasia, fibrosis was scored according to the following procedures described by Hund et al. (36). Once complete, the pronephros or head kidney was removed from the fish using sterile dissecting tools. The head kidney was chosen for gene expression analyses due to its role as an important immunological organ and its tissue density. Prior analyses confirmed that the head kidney exhibits transcriptomic responses to tapeworm infection and is associated with fibrosis in the body cavity. However, future studies should consider gene regulatory changes in the peritoneal cavity. To do so, one would first need to overcome the substantial technical challenge of obtaining a consistent biopsy of fibrotic tissue (which tends to connect many different organs in the body cavity) obtaining high-yield RNA from fibrotic tissue.

The sampled tissue was suspended in RNAlater, stored at 4 °C overnight, and then placed at −80 °C for long-term storage. Approximately 10 fish per population and treatment combination were sampled and sequenced at each of the first three time points (Days 1, 10, and 42; **Supplementary Table S1**). Day 90 represented opportunistic sampling of the remaining fish and had much lower and more variable sampling sizes; therefore, it was excluded from most analyses, except for trajectory analyses. All samples sequenced in this study were included in our previous analysis of the phenotypic patterns of fibrosis (36).

### RNA extraction, sequencing, and processing

RNA was extracted from the head kidney tissues using an Ambion MagMax-96 RNA Isolation Kit, following existing protocols (49). The extracted RNA was sent to the UT Genomic Sequencing and Analysis Facility for TagSeq Library preparation (50) and sequencing on a NovaSeq platform. All resulting reads were processed using the iRNAseq pipeline (51), which includes initial read deduplication, adaptor trimming, and quality filtering. Reads were aligned to version 5 of the UGA stickleback transcriptome assembly (52–54) using bowtie2 (55).

### Gene expression analysis

The raw data and code for all analyses described herein can be found on GitHub (https://github.com/lfuess/InjectionMS). Prior to analyses, the reads were normalized using the variance-stabilizing transformation in the R package DESeq2 (56). We then filtered expression to retain only those in the upper two quartiles of relative expression (abundant mRNAs); by reducing the number of genes considered, we sought to simplify the functional interpretation of our results. A caveat is that by excluding low-expression genes, we might overlook some differentially expressed genes that naturally have low mRNA abundance (likely including some relevant cytokines or chemokines). **Supplementary File 1** presents a more complete analysis with the top 75% expression level genes; this altered filtering approach yielded similar qualitative conclusions about the proportion of differentially expressed genes and the overlap between the differential expression of different populations and treatments.

In any tissue-level transcriptomics study, variations in mRNA relative abundance can be attributed to several biological processes. A given gene’s mRNA might be abundant (or rare) because it is upregulated or downregulated, in the sense that chromosome unpacking and transcription factors (repressors) actively increase the rate of transcription per cell. However, one must consider alternatives, such as variations in mRNA degradation rates within cells and changes in cell populations. The latter is especially important for immune organs, where cell proliferation and migration play key roles in the immune response, changing the relative abundance of different cell types. For instance, an increase in T cell numbers would increase the abundance of mRNAs primarily expressed in T cells. The existence of established immune cell markers from previous scRNAseq studies (37) helped interpret our bulk RNA-seq results (detailed below). However, formal quantitative deconvolution to calculate changes in cell population abundance and activation states is premature in sticklebacks. For instance, existing scRNA-seq annotations lack some key cell populations that appear to drop out during library preparation.

The experimental design used in this study is inherently complex, involving three different factors that can reasonably be expected to exhibit significant and biologically interesting interactive effects. Unfortunately, many RNA-seq analytical packages (i.e., DESeq2) are not well equipped to handle such multi-way interaction models. Consequently, we used a quasibinomial general linear model (GLM) to test whether each gene’s read count (proportional to the total read depth) depended on the population (SAY, GOS. RSL), treatment (PBS control vs. Alum; or PBS control vs. cestode protein in a second GLM), and sample time point. A quasibinomial GLM was specifically chosen based on two factors: 1) the proportional nature of gene expression data (evaluation of the abundance of gene sequences out of a total number of sequences) and 2) the overdispersion, which often characterizes gene expression data [although this is less severe for 3’-TagSeq Data (50)]. Additionally, a quasibinomial is a more conservative approach (yielding fewer significant effects than DESeq2), increasing our confidence in the reported findings.

Each GLM included all pairwise and three-way interactions. A significant main effect of treatment (or treatment × time) without any interactions with population (population × treatment or population × treatment × time) implies that there is a transcriptomic response to injection that is shared by the three populations. An interaction between treatment and time would highlight genes whose abundance responded to injection contents, but where this effect changed over time. A treatment × population interaction indicates genes that show population differences in response to injection (and treatment × population × time indicates genes where population differences in injection response shift over time). For the purposes of this analysis, we do not discuss the effects of population, time, or population × time, as these model terms focus on potentially constitutive population differences (or effects of age) unrelated to experimental treatment and therefore are not relevant to the questions we seek to answer. We use ‘differential expression’ (DE) to describe genes whose mRNA abundance differs between treatments (e.g., alum vs. saline), keeping in mind the caveat above that multiple processes affect mRNA abundance.

Finally, we chose to report uncorrected *p-*values instead of using a traditional correction approach for multiple comparisons. This is reflective of the fact that gene expression data are inherently highly correlated, and thus, each test is not truly independent. Thus traditional (i.e., Bonferroni-type) corrections would be overly conservative. As an alternative, we adopted a two-pronged approach. First, we used a slightly more stringent cutoff for significance (α = 0.01). Second, we tested for biologically significant signals by comparing the number of differentially expressed genes against null expectations for type I error rates. With our stringent cutoff (α = 0.01), we expect that false discovery alone (type I error) would result in approximately 130 genes (1% of the approximately 13,000 being tested with the GLMs) reaching our threshold for statistically significantly differentially expressed (DE) for a given effect. We used a binomial proportion test to check whether the number of DE genes was greater than that explained by type 1 error alone (i.e., exceeded the 1% null expectation) for each model term (treatment, treatment × time, treatment × population, and treatment × population × time).

To further validate that our pre-filtering did not omit important patterns of immune gene differential expression, we also used gene ontology (GO) enrichment terms to isolate candidate immune genes among our untested (lower 50%) genes. Candidate immune genes were identified as those with GO terms matching a set of determined keywords (**Supplementary File 2**). These genes were then screened for differential expression using the same models described above and the same approach to test for the statistical overrepresentation of differentially expressed genes.

To evaluate the similarities between DE response to alum and cestode protein, we wished to know how many genes exhibited DE in both treatments (relative to saline controls). If the two sets of DE genes are independent, with proportions x and y responding to alum and protein, respectively, the null expectation is that a proportion x ∗ y will be significant for both. We used a binomial proportion test to compare the observed overlap in DE genes against this null expectation. Furthermore, we tested for a correlation between the effect sizes (log-2 fold change, L2FC) for both treatments using Pearson’s correlation. Correlations were run on two sets of data: 1) genes that were significantly differentially expressed in one or both models for the factor of interest, and 2) all genes tested by both models.

To further investigate population differences in transcriptomic responses, we split our samples by population and ran additional binomial GLMs with the term’s treatment, time, and treatment ∗ time for alum and cestode treatments independently. We then used correlative analyses (Pearson correlations) to test for cross-population congruence in the responses of genes that were significantly differentially expressed in one or more populations using all possible pairwise comparisons.

Next, we considered the role of fibrosis in driving the observed patterns of differential expression using a third quasibinomial GLM, which tested whether the read count depended on the fibrosis score or the pairwise interaction of the fibrosis score and population. This model was run for the entire dataset (days 1–42, control, alum, and cestode protein-treated) but focused on the general correlation of fibrosis with gene expression and did not consider differences in fibrosis as a result of treatment or time of sampling. Fibrosis scores from the injected side of the fish were used (36). Significantly differentially expressed genes were compared across the three models for downstream interpretation. For all analyses, our discussion specifically focused on those genes identified as putative immune genes based on updated annotations associated with version 5 of the UGA stickleback transcriptome assembly (52–54).

As a coarse approach to determine the extent of the variance observed in our three main statistical models (alum, cestode protein, and fibrosis) due to changes in cell type abundance, we applied deconvolution approaches using previously identified immune cell type markers (37). Markers for eight major head kidney cell types were used: antigen-presenting cells, B cells, erythrocytes, fibroblasts, hematopoietic cells, natural killer cells, neutrophils, and platelets. Following the methods detailed in Fuess and Bolnick (37), we first tested for overrepresentation of cell type markers generally among each list of significantly differentially expressed genes associated with our model terms of interest (treatment, treatment × time, treatment × population, and treatment × time × population for alum/cestode protein models; fibrosis and fibrosis × population for fibrosis model). We then tested for the overrepresentation of cell-type-specific markers in those groups where a general overrepresentation of cell-type markers was identified. All overrepresentation tests were conducted using χ-squared tests on each list of genes independently. Finally, we used 1-sample proportion tests to test for the significance of expression patterns (i.e., greater representation of up- or downregulation than expected) in groups of markers that were significantly overrepresented.

Finally, we conducted a trajectory analysis to evaluate the temporal progression of transcriptomic responses to the immune challenge in each population, as described by Torres et al. (57). To do so, we generated discriminant analysis of principal components (DAPC) axis scores for each individual using all combinations of population, treatment, and time point as grouping variables. Analyses were conducted on a reduced set of genes that were identified as significant (α = 0.01) for at least one of our model terms. We then calculated the mean DAPC score for each time point in each population. Control fish represented the unactivated resting state, a substitute for time zero gene expression profiles. We then calculated the multivariate vectors connecting each time point to the successive time point for a given population. For each population, we can then represent the ‘arc’ of the immune response as a series of end-to-end vectors in DAPC space, connecting successive time points. In principle, these arcs should return to their resting state if full recovery is achieved (57), providing a visual representation of immune ‘resilience.’ Note that individual gene trajectories might be quite different from the transcriptome-wide consensus trends measured by DAPC.

## Results

### Differential expression results summary

**Figure 1:**
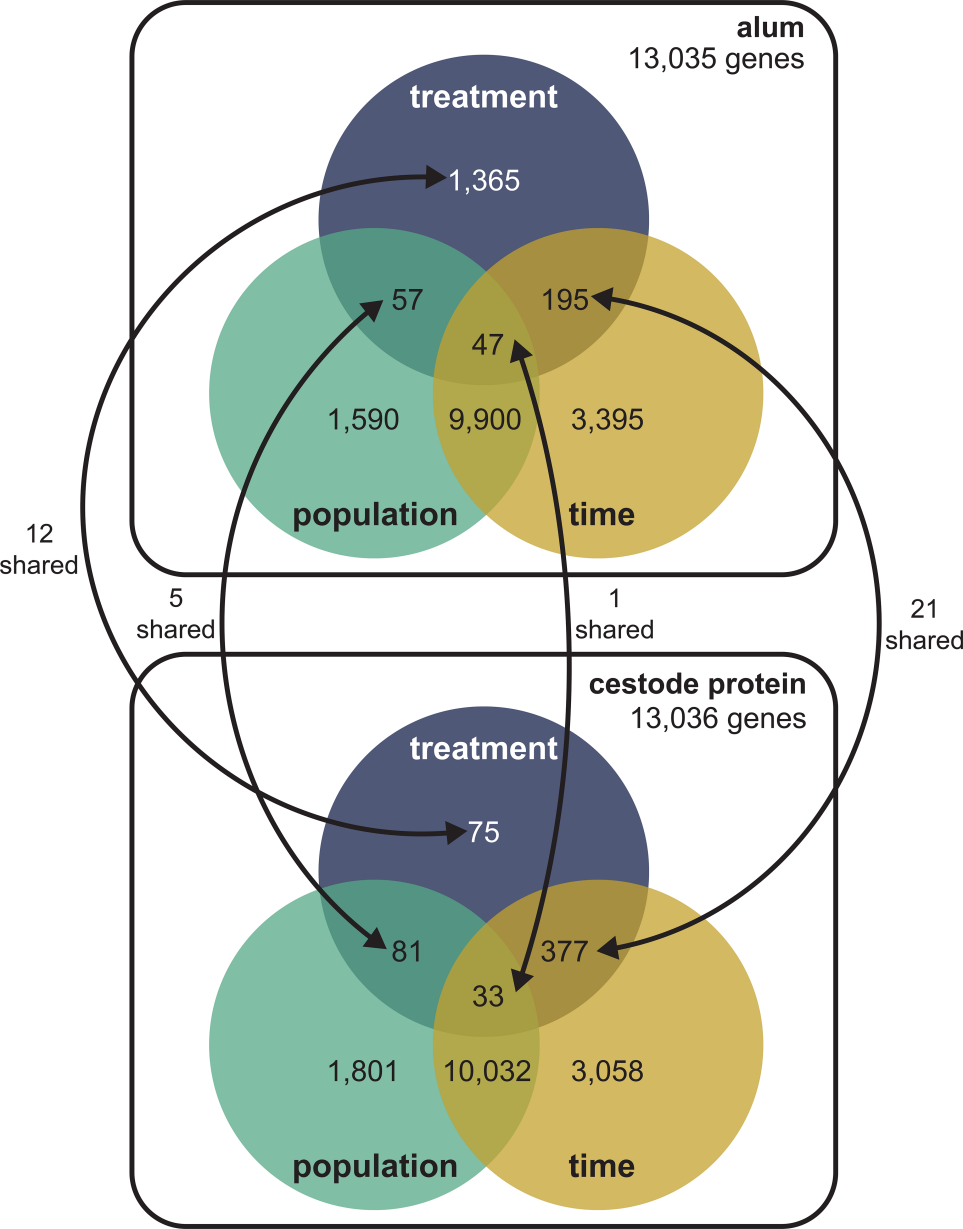
Conceptual summary of differential expression results for alum and cestode protein models. Total number of genes tested for each are displayed underneath the model title. Numbers displayed represent total differentially expressed genes for each main effect or interaction effect (where circles overlap). Arrows between the two diagrams indicate shared significant genes across both models. For the purposes of this study, we focused on effects (main and interactive) associated with treatment only.

Differential expression modeling using quasibinomial GLMs revealed the targeted effects of alum and cestode protein injections on the transcriptome composition of the stickleback head kidney (**Figure 1**; **Supplementary File 3**). The main effects of alum injection were most significant, inducing changes in 1,365 genes out of 13,035 tested (approximately 10.5%, far greater than our null expectation of 1%; binomial proportion test; *p <*0.001). Nearly all of these genes (1,347 total) were significantly differentially expressed as a result of the main effect of alum only (no interaction terms significant), indicating the strong effects of alum independent of population or timing. Of these genes, 55 were significant following Bonferroni correction at an FDR of 10% (seven immune functioning, three related to T cells: *gpnmb*, *lck*, and *bcl11b*). An alum × time interaction affected 195 genes, significantly greater than our null expectation (binomial proportion test; *p <*0.001). Two of these genes were significant following Bonferroni correction at an FDR of 10% (one immune: *pim1*). This interaction indicates that there is a time course of genes that are transiently expressed (or repressed) in response to treatment, similarly for all populations. In contrast, alum × population interaction (57 genes) and three-way interactions (47 genes) both induced less differential expression than expected under false discovery rate expectations alone (binomial proportion test; *p <*0.001). Furthermore, none of these genes were significant following Bonferroni correction; therefore, we cannot confidently infer any population-level differences in the transcriptome response to alum at the level of individual genes.

In contrast to alum, the number of significantly differentially expressed genes associated with the main effects of cestode protein treatment (only 75 out of 13,036 tested, or 0.58%) was lower than the false discovery rate expectations (*p <*0.001). Most of these 75 genes exhibited no interaction between treatment and time (or population). However, we did observe a large number of genes that changed in response to cestode protein over time; 377 genes were significantly dependent on a treatment × time interaction, greater than false discovery alone could yield (*p* < 0.001). This suggests that cestode protein treatment induces a dynamic response over time rather than the persistent effects that characterize responses to alum. All other terms, including treatment × population interaction (81 genes) and three-way treatment × time × population interaction (33 genes), induced significantly less differential expression than predicted under null expectations (*p <*0.001). No genes differentially expressed in our cestode protein model were significant after Bonferroni correction (FDR of 10%).

Finally, our GLM analysis of expression associations with fibrosis yielded 2,888 genes whose expression was associated with fibrosis (regardless of treatment and time) and 389 genes that were significantly dependent on the interaction of fibrosis and population (**Supplementary File 3**). Both exceeded the false discovery null expectations (*p* < 0.001). Of the genes with a significant main effect of fibrosis, 146 were also significant for the interaction of fibrosis and population. Finally, 648 genes were significantly dependent on the fibrosis score following Bonferroni correction at an FDR of 10%, whereas only one was significant after Bonferroni correction for the fibrosis × population interaction. These results indicate a strong transcriptomic signature of the general fibrosis response, regardless of treatment type, time, or, to some extent, population. These analyses rely on a subset of genes in the transcriptome (the top two quartiles by mean proportional expression). Naturally, the number of differentially expressed genes reported above would be larger if we set a lower threshold (e.g., including more rarely expressed genes), and some functionally relevant genes (e.g., some low-expression cytokines or chemokines) may be overlooked. However, the overall patterns of differential expression and (dis)similarities among populations, time points, and treatments were retained with a broader set of genes (see **Supplementary File 1**). Furthermore, targeted analysis of immune genes in the lower two quartiles of mean proportional expression revealed patterns consistent with those described above and did not identify any novel patterns of differential expression among functionally relevant gene groups (**Supplementary File 2**).

### Shared responses to alum and cestode protein injection

**Figure 2:**
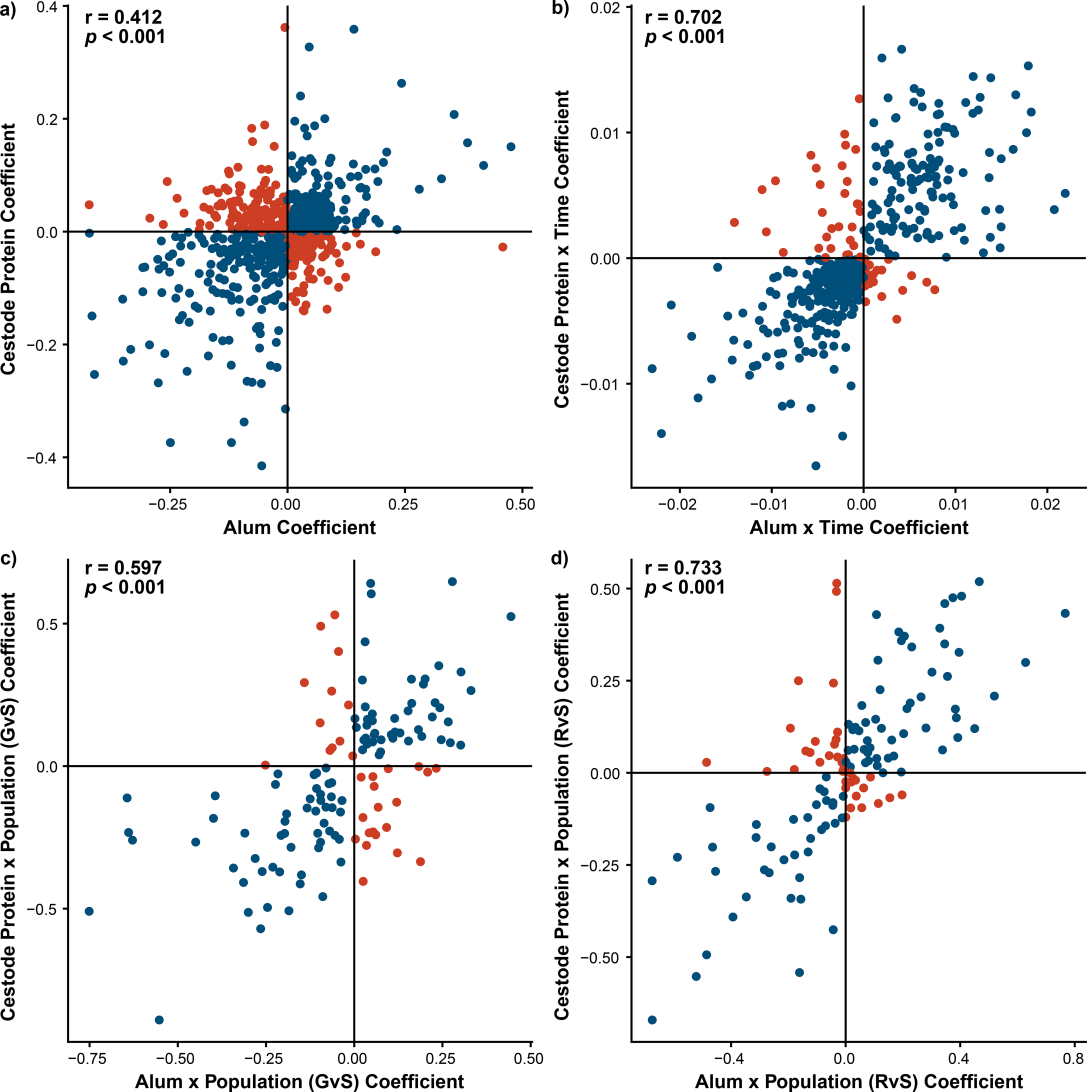
Responses to treatment are largely congruent across alum and cestode protein. Scatterplots display relationship between responses to **(A)** treatment main effects, **(B)** treatment by time effects and **(C, D)** treatment by population effects across alum and cestode protein treatments. All points represented a gene which is significantly differentially expressed in at least one of the two models (alum or cestode protein) Points are colored based on the relationship of the coefficients for each model, wherein blue dots indicate congruence across alum and cestode protein effects and red points indicate divergence. Pearson correlation results are displayed for each comparison.

When comparing differential expression responses to alum and cestode protein across main and interaction effects, we observed limited patterns of conserved responses (**Figure 1**). Only 12 genes were differentially expressed (uncorrected *p* < 0.01) in response to both treatments, which were not significantly greater than the null expectation of approximately eight genes (*p* = 0.179). However, counting the overlap of significant genes can be misleading when the power is moderate or low. More importantly, the responses to alum and cestode protein were largely positively correlated, both when considering only significantly differentially expressed genes (**Figure 2A**) and when considering all genes tested in both analyses (**Supplementary Figure S2A**). This positive correlation confirms that there is a shared basis for the transcriptomic response to alum and cestode proteins, which is not detectable by examining the patterns of individual genes alone. This shared basis of alum and protein response is more obvious when we consider the similarities in treatment × time and treatment × population effects. A total of 21 genes (approximately six expected) exhibited significant DE treatment × time interaction for both alum and protein treatments, more than false discovery expectations (test of equal proportions; *p <*0.001). The effect sizes for alum and protein were also positively correlated, whether we considered only the subset of differentially expressed genes (**Figure 2B**) or all genes (**Supplementary Figure S2B**). Finally, five genes exhibited treatment × population effects for both alum and protein injections (more than the <1 null expectation; *p <*0.001), again demonstrating strong congruence across treatments (**Figures 2C, D**; **Supplementary Figures S2C, D**). Only a few of these shared genes across all effects were related to immunological processes, with no clear trends in the shared pathways (**Supplementary Figure S3**).

**Figure 3:**
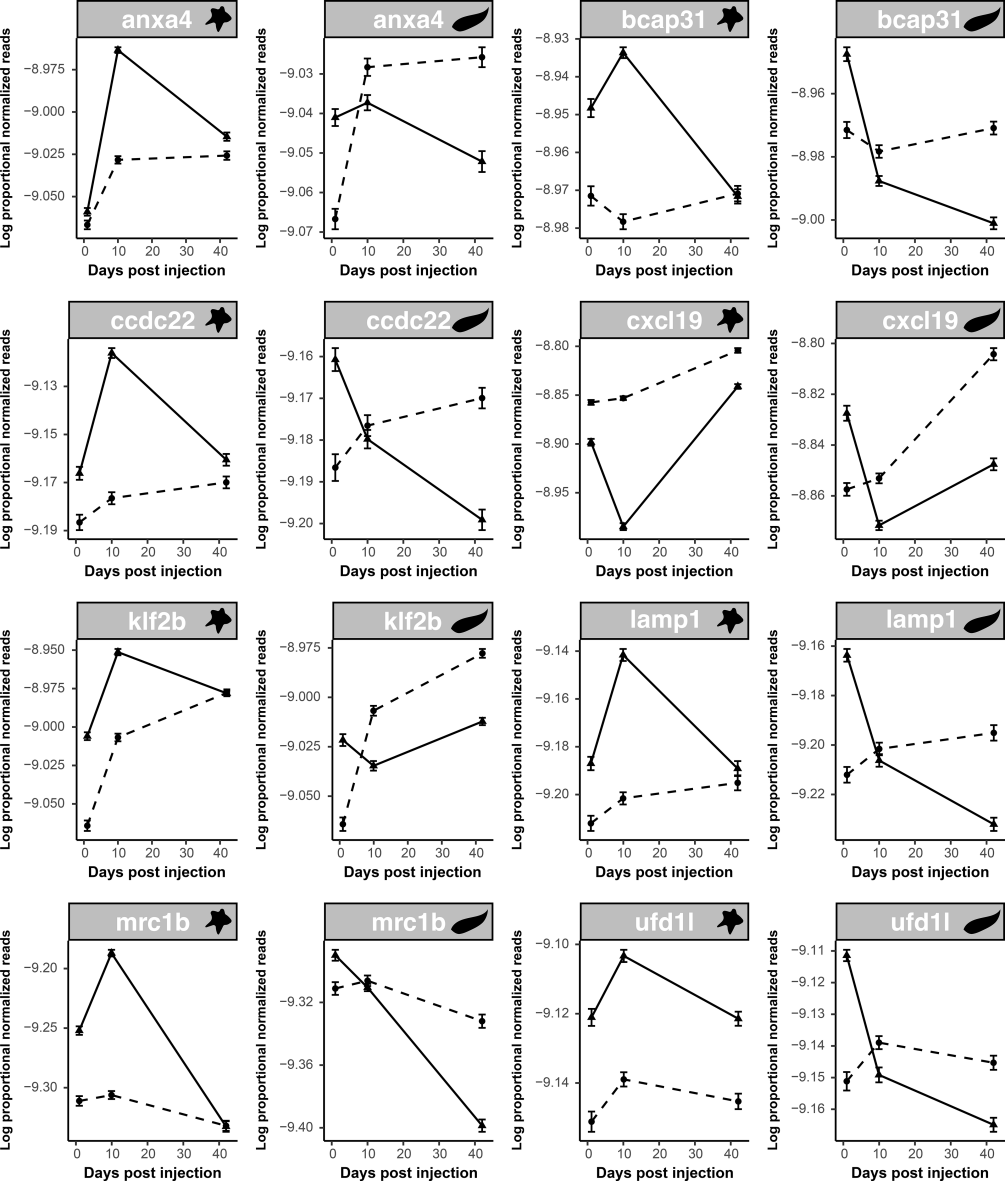
Responses to cestode protein are more dynamic over time when compared to alum. Line plots display log proportional normalized read count values of putative immune genes which were differentially expressed as a result of both alum main effects and cestode protein by time effects. Plots show trajectories of treatment and control groups over time. Plots are paired with alum response on the left (indicated with star icon) and cestode protein response on the right (indicated with worm icon). Dotted lines indicate control values whereas solid lines indicate treatment values. As there were no significant population effects, lines are shown for all fish within a treatment combined across populations.

**Figure 4:**
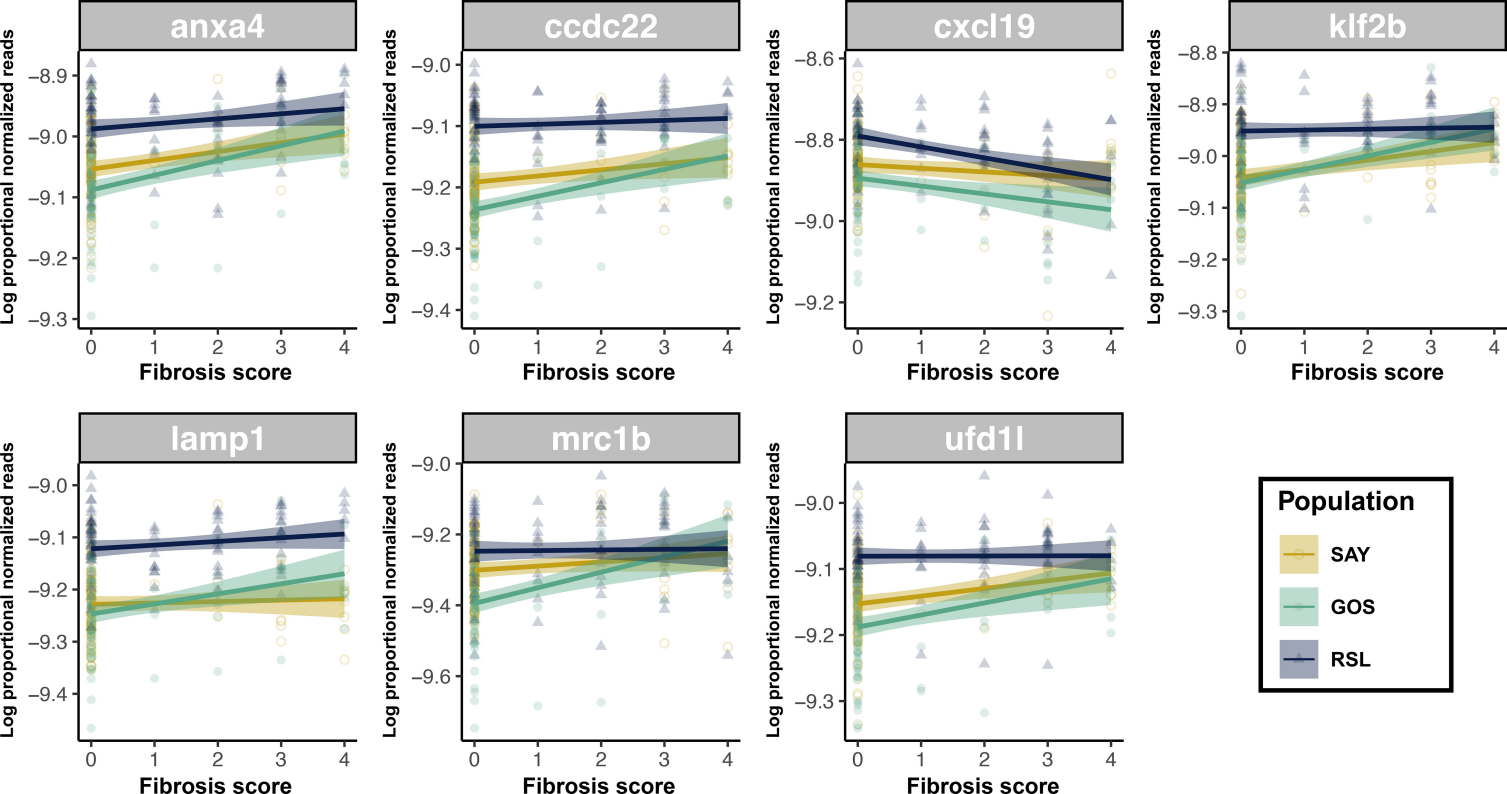
Genes which were more dynamic over time in response to cestode protein, but also responsive to alum, are associated with fibrosis. Scatter plot display associations between log proportional normalized count values and fibrosis scores for genes which were differentially expressed as a result of both alum main effects and cestode protein by time effects. Points and lines are colored by population; lines represent population-specific linear models with 95% confidence intervals shaded. Data is shown and linear model lines drawn based on all treatments and timepoints combined.

Genes that exhibit DE in response to the main effect of treatment also exhibit DE in the interaction terms. Biologically, this means that the genes that generally respond to antigen (or adjuvant) injections are also the genes that show among-population (or among-time point) differences in this response. This observation provides strong evidence that this DE is biologically real and not a type II error. We tested for overlap in DE genes between all possible pairwise combinations of statistical effects, for example, comparing the main effect of alum versus alum × population or alum × time. We also compared the main effects and interaction effects of different injection treatments. Of these comparisons across treatment models (summarized in **Supplementary Table S2**), surprisingly, the comparison of alum main effects and cestode protein × time was the only pairwise comparison in which the number of overlapping significant genes exceeded null expectations. A total of 54 genes were differentially expressed as a result of both alum main effects and cestode protein × time interactions, significantly greater than the expected 39 genes (*p* = 0.0200). This observation shows that the set of genes that react persistently to alum overlaps substantially with the set of genes that react transiently to cestode protein. Eight of these genes have immunological functions, all but one of which were consistently upregulated in response to alum. In contrast, all these genes in cestode protein-treated fish started as upregulated on day 1 (compared to the PBS control), but by day 42, they were downregulated relative to the controls (**Figure 3**). This suggests that within this core set of genes responding to both treatments, there is a compensatory downregulation unique to cestode protein responses. Furthermore, seven out of eight of these genes were significantly correlated with fibrosis, two of which were also differentially correlated across populations (*klf2b* and *mrc1b*; **Figure 4**). All of these genes were also significantly differentially expressed as a result of population in the fibrosis model, and all were significantly higher in RSL than in GOS and SAY (Tukey *post-hoc*; **Supplementary Table S3**; **Supplementary Figure S4****).** Furthermore, for those genes with fibrosis × population effects, neither was significantly positively associated with fibrosis in the RSL. Specifically, *klf2b* was significantly positively correlated with fibrosis in the GOS and SAY populations only (population-specific quasibinomial GLM; **Supplementary Table S4**), and *mrc1b* was significantly positively associated with fibrosis in the GOS population only (population-specific quasibinomial GLM; **Supplementary Table S4**).

### Unique responses to alum and cestode protein injection

**Figure 5:**
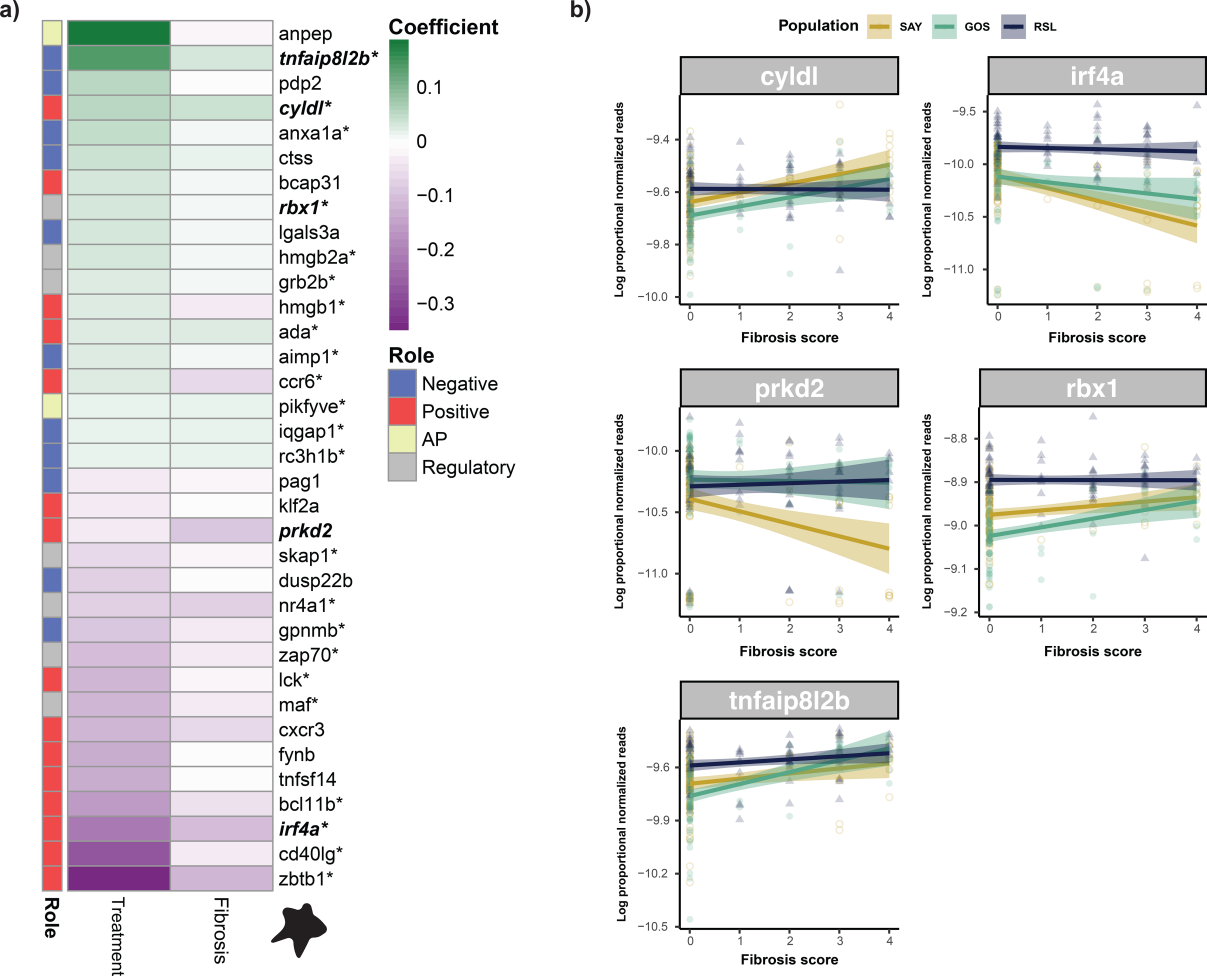
Summary of alum main effects and fibrosis effects on the subset of T cell related genes which are uniquely responsive to alum. **(A)** heatmap displaying coefficient values for alum and fibrosis main effects for our unique alum genes which are associated with T cell function; The annotation bar summarizes the relationship of each gene to T cell function (POS, positive effect on T cell functioning; NEG, negative or suppressive effect on cell functioning; AP, antigen presentation; REG, contributes to the general regulation of cell function in what could be positive and/or negative ways). * indicates significance for fibrosis main effect. Bolded and italicized gene names indicate those which also had a significant effect of fibrosis by population. **(B)** Scatter plot displaying associations between log proportional normalized read values and fibrosis scores for T cell genes which were differentially associated with fibrosis across population. Points and lines are colored by population; lines represent population-specific linear models with 95% confidence intervals shaded. Plots include both alum and cestode protein data. In summary, genes associated with T cell activity show largely congruent responses to alum and associations with fibrosis, but those with population specific responses are more associated with fibrosis in GOS and SAY fish, but constitutively highly expressed in RSL fish. Data is shown and linear model lines drawn based on all treatments and timepoints combined.

Of the genes responsive to the main or interactive effects of alum, 110 were identified as putative immune genes, 46 of which (nearly half) were related to T cell function and antigen presentation. Nearly all these genes were uniquely responsive to alum treatment or alum-by-time interactions. A total of 35 genes that may be related to T cell function were significantly differentially expressed depending on the alum main effects, with complex associations between alum treatment and activation/function of genes associated with a wide variety of T cell subtypes (**Figure 5A**). Of these, 25 were also associated with either fibrosis or the interaction of fibrosis × population; the effect sizes of gene responses to alum treatment were positively correlated with the gene responses to fibrosis (Pearson correlation; *p <*0.001, r = 0.666; **Figure 5A**). This indicates that the transcriptional basis of fibrosis is driven by a T cell response, which also responds to alum. All but three of these genes (*nr4a.1, rc3h1b*, and *skap1*) were also significantly differentially expressed as a result of the main effect of population in the fibrosis model (**Supplementary Table S3****).** Furthermore, 18 of these were significantly differentially expressed in RSL compared to GOS and SAY, all but three of which were constitutively expressed at higher levels in RSL (Tukey *post-hoc*; **Supplementary Table S3**). Furthermore, of the five genes with a fibrosis × population interaction effect, none were significantly associated with fibrosis in the RSL, and four of five were significantly associated with fibrosis in both GOS and SAY (population-specific quasi-binomial GLM; **Supplementary Table S4**). From these population effects, we inferred that the T cell responses associated with fibrosis are population-specific.

**Figure 6:**
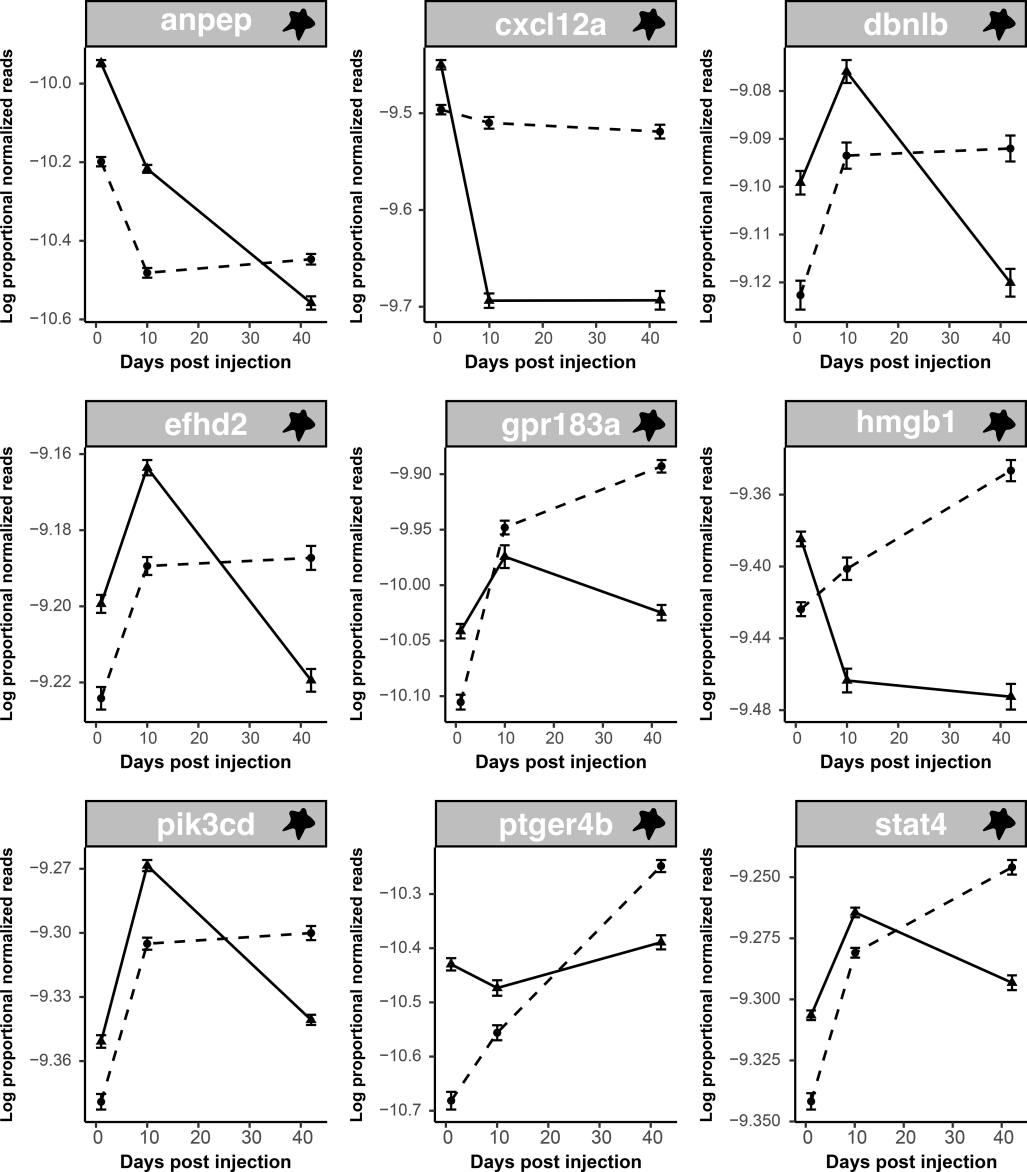
Some T cell genes which are responsive to alum display dynamic patterns of regulation; most start upregulated and end up suppressed. Line plots display log proportional normalized read count values of uniquely alum-responsive T cell genes which were differentially expressed in response to alum over time. Plots show trajectories of treatment and control groups over time. Dotted lines indicate control values whereas solid lines indicate alum treatment values. As there were no significant population effects lines are shown for all fish within a treatment combined across populations.

Several T cell-associated genes were also differentially expressed in response to alum over time, all of which were upregulated on day 1 (relative to the PBS control) but downregulated by day 42 (**Figure 6**). This upregulation followed by downregulation is indicative of an activation stage followed by compensatory repression, similar to those noted previously. Only two of these nine genes were affected by fibrosis, both negatively. Finally, of the 102 alum-responsive genes with alum × population interaction effects, only four have roles in immunity, three of which are related to T cell function. Each of these is differentially responsive to treatment in GOS compared to RSL and SAY fish (**Supplementary Figure S4**). None of these overlapped with fibrosis.

**Figure 7:**
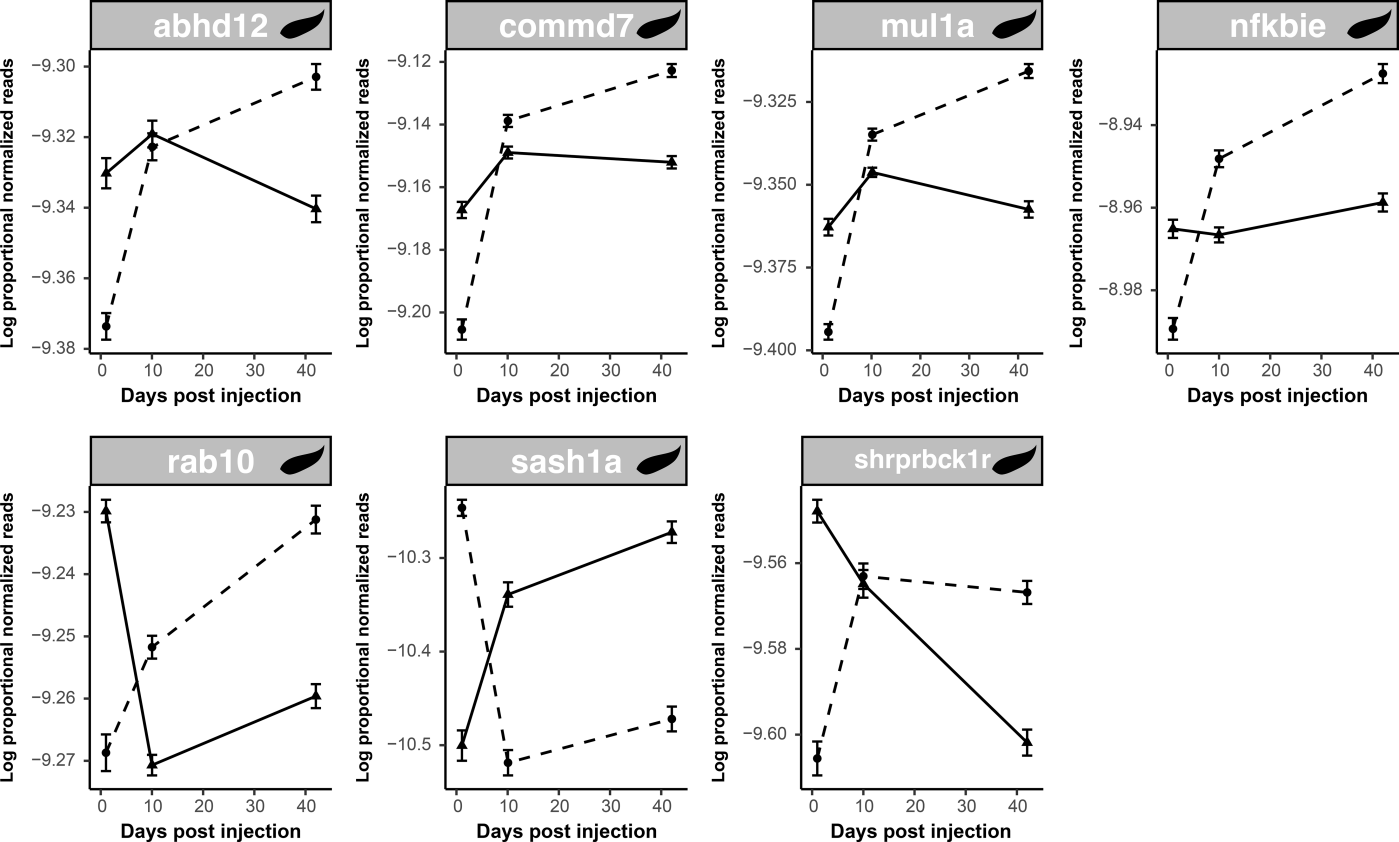
Many NF-κB genes which are responsive to cestode protein display dynamic patterns of regulation; most start upregulated and end up suppressed. Line plots display log proportional normalized read count values of uniquely cestode protein-responsive NF-κB genes which were differentially expressed in response to cestode protein over time. Plots show trajectories of treatment and control groups over time. Dotted lines indicate control values whereas solid lines indicate cestode protein treatment values. As there were no significant population effects lines are shown for all fish within a treatment combined across populations.

When considering unique responses to cestode proteins, we observed an abundance of genes involved in activating and regulating the function of the key immune transcription factor NF-kB. Thirteen of the 39 identified immunological genes affected by the main or interactive effects of cestode protein are related to NF-κB functioning (nine unique to cestode protein). It should be noted that numerically more NF-κB genes responded to alum than to the cestode protein; however, the proportion of NF-κB genes was much higher among cestode protein genes, especially when considering unique genes. Of the unique genes, seven were significantly differentially expressed in response to cestode protein over time; all but one of these genes began to be upregulated at day 1 (compared to the PBS control) and were downregulated by day 42 (**Figure 7**). This suggests a cycle of gene activation and repression. Only three of these overlapped with fibrosis; all three were also significant for population and were expressed at higher levels in RSL than in GOS/SAY (Tukey *post-hoc*; **Supplementary Table S3****).** Furthermore, *mul1a* and *nfkbie* were negatively associated with fibrosis regardless of the population, while *commd7* was positively associated with fibrosis in the GOS and SAY only (population-specific quasi-binomial GLM; **Supplementary Table S4**).

**Figure 8:**
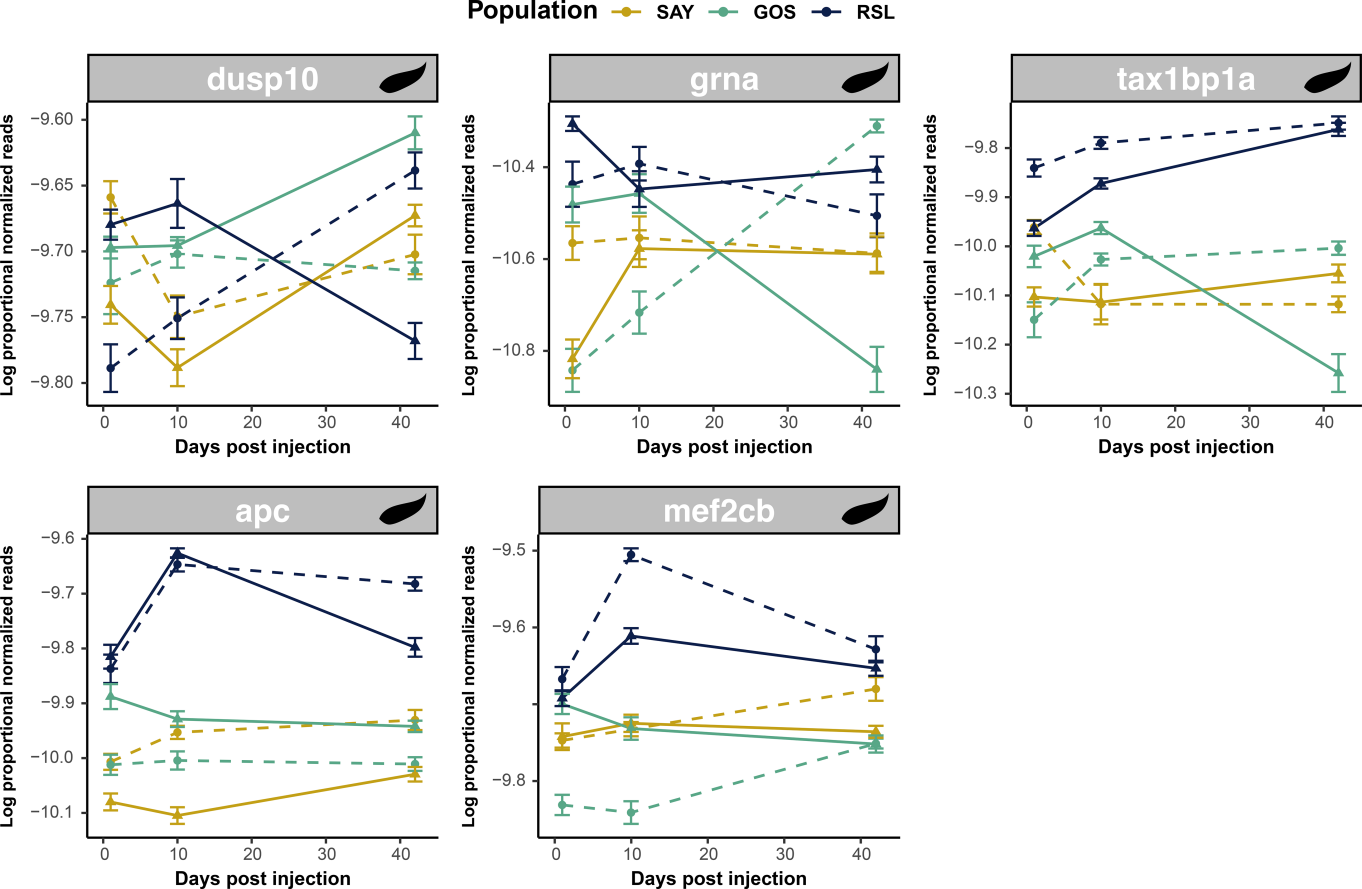
Inflammatory responses to cestode protein are population specific. Line plots display log proportional normalized read count values of uniquely cestode protein-responsive inflammation genes which were differentially expressed in response to cestode protein across population. Plots show trajectories of treatment and control groups over time. Lines are colored based on population. Dotted lines indicate control values whereas solid lines indicate cestode protein values. Genes are sorted based on effects—the top row displays genes with significant three-way interactions (treatment by population by time) whereas the bottom row shows genes with treatment by population effects.

Related to NF-κB functioning, we also observed strong enrichment of inflammation-related genes, some of which overlapped with our NF-κB genes, as expected. Twelve of the 39 differentially expressed genes related to immune-related cestode proteins were associated with inflammatory processes (seven unique to cestode proteins). Of the unique inflammation-related genes associated with cestode proteins, five were affected by the interaction of population and cestode protein treatment effects or the three-way interaction of all effects (treatment, population, time; **Figure 8**). All five were divergent in GOS fish compared to RSL and SAY fish. Only one of these, dusp10, was also negatively associated with fibrosis.

**Figure 9:**
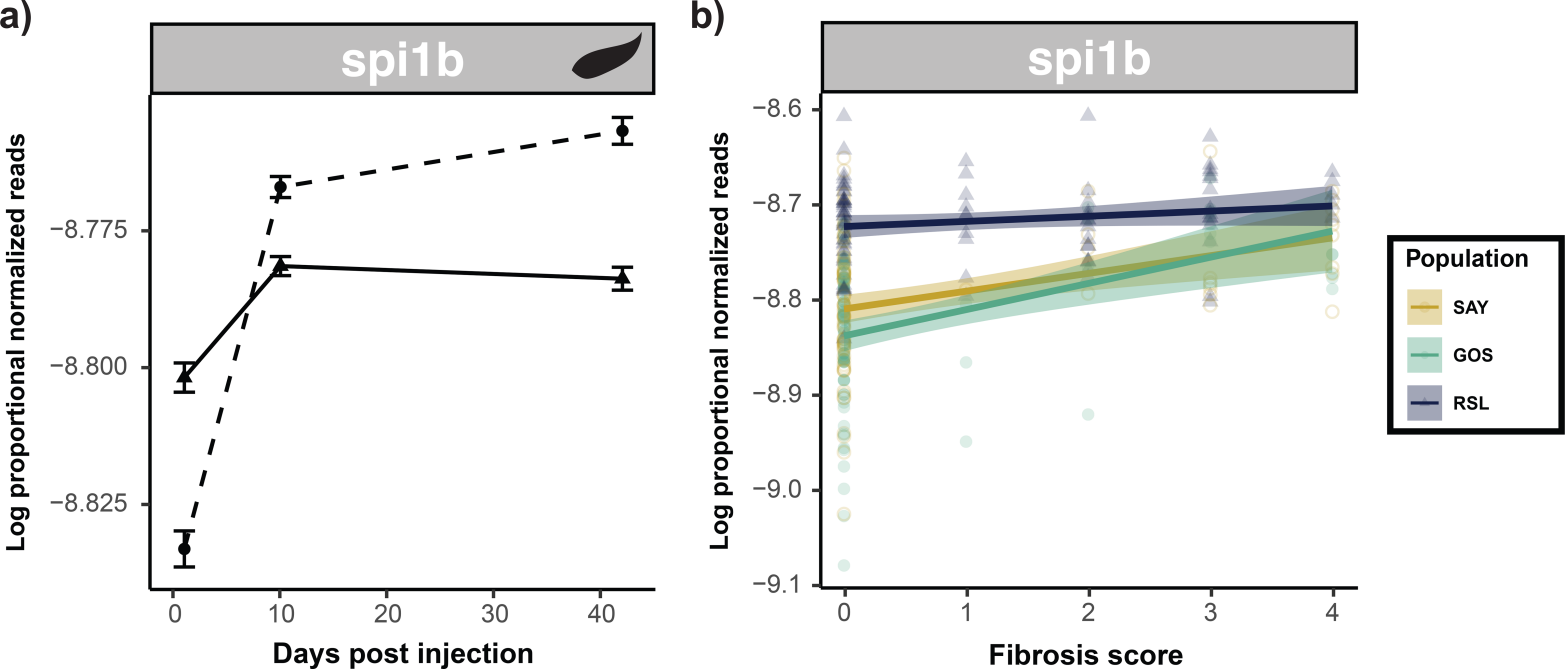
Summary of differential expression of putative stickleback fibrosis regulator gene *Spi1b* in response to cestode protein treatment and its effect on fibrosis. *Spi1b* is dynamically responsive to cestode protein treatment (initially upregulated, but eventually downregulated) and positively associated with fibrosis in GOS and SAY only. In RSL fish it is constitutively highly expressed. **(A)** Line plots displaying log proportional normalized read count values trajectories of treatment and control groups over time. Dotted lines indicate control values whereas solid lines indicate cestode protein values. As there were no significant population effects lines are shown for all fish within a treatment combined across populations. **(B)** Scatter plot displaying associations between log proportional normalized read count values and fibrosis scores. Points and lines are colored by population; lines represent population-specific linear models with 95% confidence intervals shaded. Data is shown and linear model lines drawn based on all treatments and timepoints combined.

Finally, the putative fibrosis regulator gene, *spi1b* (58), was also significantly differentially expressed in the cestode protein group only and was differentially associated with fibrosis across populations (**Figure 9**). *Spi1b* was initially upregulated in response to the cestode protein but was downregulated by day 42. Notably, *spi1b* was significantly differentially expressed across populations (highest in RSL; Tukey *post-hoc*; **Supplementary Table S3**) and was strongly positively associated with fibrosis in GOS and SAY fish only (population-specific quasibinomial GLM; **Supplementary Table S4**). This differential effect among populations is consistent with the inducible *spi1b* expression in GOS and SAY, which changed to constitutively high expression in the most fibrosis-prone RSL population.

### Changes in cell type specific markers

The advent of single-cell RNA sequencing techniques has resulted in the identification of novel cell-type-specific transcriptomic markers and the development of applications for their use in improving the interpretation of bulk RNA-seq data. We leveraged existing head kidney cell-type-specific markers (37) to assess whether some of the patterns described herein could be explained by changes in cell type abundance (as opposed to transcriptional changes). Generally, genes differentially expressed as a result of alum main effects (χ^2^ = 45.2, *p <*0.001), cestode protein × time interactions (χ^2^ = 4.09, *p* = 0.0432), and fibrosis main effects (χ^2^ = 79.15, *p <*0.001) were statistically significantly enriched for head kidney cell type-specific markers. Further investigation of cell-type-specific markers revealed unique patterns of response to each of these model terms (**Table 1**). Specifically, in response to alum, we observed a significant overrepresentation of antigen presenting cell, B cell, hematopoietic cell, and natural killer cell makers. Furthermore, antigen-presenting cell, B cell, and hematopoietic cell-associated genes were almost exclusively upregulated, whereas natural killer cell-associated genes were almost exclusively downregulated. In contrast, antigen-presenting cells were the only specific cell type overrepresented among cestode protein × time interaction genes, and all of the significant DEGs associated with this cell type were significantly downregulated in response to this treatment (i.e., decreasing through the experiment). Finally, the patterns associated with fibrosis were highly similar to those associated with alum. Antigen-presenting cell, B cell, hematopoietic cells and neutrophil markers were overrepresented and overwhelmingly positively associated with fibrosis.

**Table 1:** χ^2^ and proportion statistical testing for overrepresentation of specific cell type markers among lists of differentially expressed genes associated with model terms which were identified as significantly enriched for head kidney cell type markers generally.

Model (Term)	Cell type	χ^2^ test		Prop test		
		χ^2^	P-value	χ^2^	P-value	Enriched direction
Alum (treat)	APC	30.65	<0.001**	5.04	0.025*	**upregulation**
	B cell	17.13	<0.001**	0.842	0.358	n.s.
	Erythrocyte	0.181	0.671	n.s.	n.s.	n.s.
	Fibroblast	0.100	0.752	n.s.	n.s.	n.s.
	HC	34.98	<0.001**	12.5	<0.001**	**upregulation**
	NKC	12.87	<0.001**	4.17	**0.0412**	**downregulation**
	Neutrophil	6.46E−5	0.994	n.s.	n.s.	n.s.
	Platelets	0.298	0.585	n.s.	n.s.	n.s.
CP (treatment × time)	APC	21.82	<0.001**	7.111	0.0076*	**downregulation**
	B cell	0.277	0.599	n.s.	n.s.	n.s.
	Erythrocyte	n.a	n.a	n.a	n.a	n.a
	Fibroblast	n.a	n.a	n.a	n.a	n.a
	HC	3.11E−29	1	n.s.	n.s.	n.s.
	NKC	n.a	n.a	n.a	n.a	n.a
	Neutrophil	5.63E−29	1	n.s.	n.s.	n.s.
	Platelets	0.0001	0.975	n.s.	n.s.	n.s.
Fibrosis (main)	APC	20.70	<0.001**	23.361	<0.001**	**upregulation**
	B cell	20.26	<0.001**	16.46	<0.001**	**upregulation**
	Erythrocyte	0.204	0.652	n.s.	n.s.	n.s.
	Fibroblast	0.481	0.488	n.s.	n.s.	n.s.
	HC	15.37	<0.001**	16.41	<0.001**	**upregulation**
	NKC	1.85	0.174	n.s.	n.s.	n.s.
	Neutrophil	37.63	<0.001**	57.14	<0.001**	**upregulation**
	Platelets	0.696	0.404	n.s.	n.s.	n.s.

Each test was run independently. APC, antigen presenting cell; HC, hematopoietic cell; NKC, natural killer cell. Bold font indicates significant associations. * p < 0.05; **p < 0.001.

### Population specific responses

Multivariate analyses of the transcriptome can reveal broader coordinated changes across many genes. One multivariate approach is to compare the entire vector of differential expression (DE) effect sizes for all genes between populations. This transcriptome-wide approach can reveal broader-scale differences in immune responses than those observed with individual genes. Correlative analyses comparing population-specific responses to alum and cestode proteins across populations confirmed the general conservation of responses to both alum and cestode proteins (**Table 2**). That is, in general if a gene is up- (or down-) regulated in response to alum (or cestode protein) in SAY fish, it is likely to change in a similar direction and magnitude in GOS or RSL lake stickleback. The exception was when we compared the two lake populations’ responses to cestode protein (GOS vs. RSL): for this one contrast, the correlation in DE values was negative rather than positive. Genes that are upregulated in the GOS tend to be downregulated in the ROS and vice versa. Genes with divergent cestode protein responses across the GOS and RSL included the antiviral gene *mxb*, complement gene *c7*, and inflammatory genes *arel1*, *cdo1*, and *dpf2* (**Supplementary Figure S5**; **Supplementary File 4**).

**Table 2:** Pearson correlation results comparing treatment and treatment by time coefficients across population-specific models for both alum and cestode protein.

Comparison	Coefficient	DEG only analysis				All genes analysis		
		r	p	Genes tested	Shared DEGs	r	p	Genes tested
GOS vs. RSL	Alum Main	**0.446**	<0.001**	812	35	**0.215**	<0.001**	11,770
	CP Main	−**0.217**	0.0118*	134	2	**-0.112**	<0.001**	11,817
	Alum × Time	0.066	0.530	93	0	**0.059**	<0.001**	11,770
	CP × Time	0.120	0.0596	247	0	**0.172**	<0.001**	11,817
GOS vs. SAY	Alum Main	**0.418**	<0.001**	883	44	**0.282**	<0.001**	12,460
	CP Main	−0.089	0.277	153	0	**-0.044**	<0.001**	12.480
	Alum × Time	**0.214**	<0.001**	247	0	**0.120**	<0.001**	12,460
	CP × Time	**0.275**	<0.001**	292	1	**0.274**	<0.001**	12.480
RSL vs. SAY	Alum Main	**0.241**	<0.001**	394	21	**0.158**	<0.001**	11,919
	CP Main	**0.171**	0.0317*	158	0	**0.046**	<0.001**	11,918
	Alum × Time	**0.230**	<0.001**	251	0	**0.185**	<0.001**	11,919
	CP × Time	0.152	0.0716	141	1	**0.199**	<0.001**	11,918

Correlations were run only on genes that were tested in both population models (i.e., in the top two quartiles of expression for both models). Two sets of correlations were run, one on only genes that were differentially expressed in one or both of the populations being compared, and one using all genes that fit the criteria. The number of genes tested in both analyses is noted, as are the total numbers of shared differentially expressed genes. Bold font indicates significant associations. * p < 0.05; **p < 0.001.

**Figure 10:**
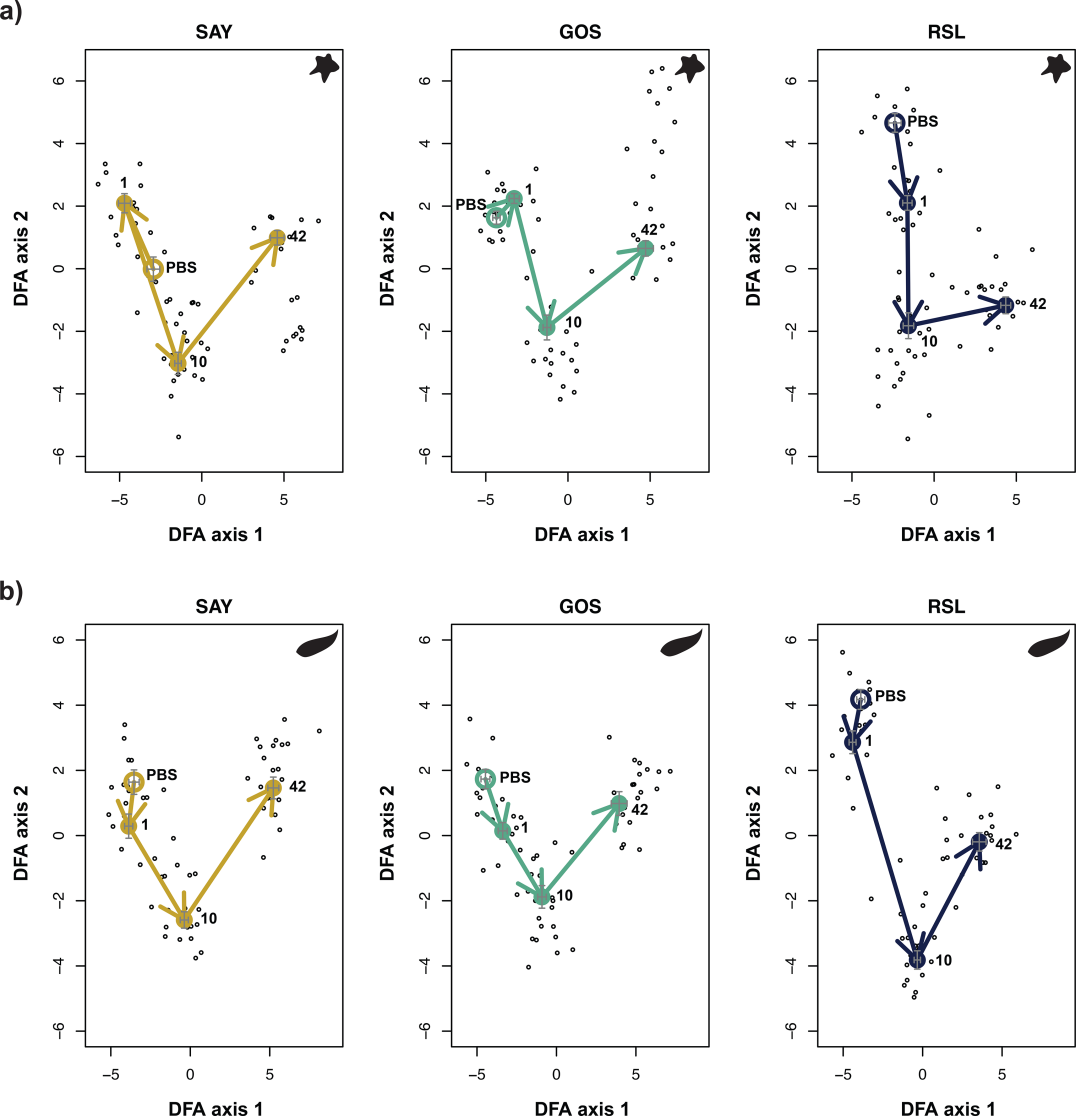
Trajectory plot of response to **(A)** alum and **(B)** cestode protein for each population independently based on genes which were significant for treatment or any interaction term including treatment across both alum and cp (2,230 total). Closed circles indicate centroids for treated fish at each time point, whereas open circle indicates centroid for control fish. Arrows indicate trajectory between time points. Crosses indicate relative spread of data points at each time point. Axes are not standardized across plots for simplified visualization. Plots show relatively similar trajectories through day 42.

Another multivariate approach developed by (57) uses ordination to summarize the variation in gene expression among individuals and track the temporal progression of transcriptomic changes. The initial direction of multivariate change after infection (or injection) indicates the early set of genes activated to drive an immune response. Later vectors reveal sets of genes driving downregulation and recovery, which could either lead to a new resting state (e.g., chronic inflammation) or return to the original resting state. To what extent do stickleback populations show parallel or divergent vectors? Trajectory analyses revealed clear between-population differences in response to treatment, which were most notable when considering cestode protein. Trajectories across the first 42 days of the experiment were largely similar for both populations and treatment types (**Figure 10**). Furthermore, no return to the PBS control state was observed in any group. However, when including data from day 90 (which had a significantly smaller sample size), we observed differences in the trajectory patterns across our three populations and treatment types (**Supplementary Figure S6**). From day 42 to day 90, RSL fish only showed some movement back towards the PBS control baseline, with stronger movement in response to cestode protein compared to alum. In contrast, SAY and GOS continued to move away from the PBS control marker, with the most pronounced movement observed in the SAY response to alum and GOS response to cestode protein. This result is consistent with the previously published observation that RSL fish partially recover from fibrosis, whereas GOS and SAY do not. Thus, trajectory analyses mostly indicate parallel responses early after injection and divergence mostly in the recovery phase. Note that because trajectory analysis uses the ordination of multivariate transcriptome data, individual genes might exhibit patterns of recovery that are obscured by transcriptome-wide summaries.

## Discussion

Parasites exert immense selective pressure on their hosts and are widely considered a key driver of host diversification and evolutionary novelty (59, 60). While robust evidence suggests that these patterns extend to the evolution of the immune system (13–15), many gaps still exist in our understanding of the mechanisms controlling the evolutionary origins of defense responses to novel parasites. One approach to address these gaps is to compare the transcriptomes of wild host populations that have acquired a new parasite with those that lack the new parasite. In a wide variety of species, recent biological invasions of parasites expanding into formerly naïve host populations have driven the rapid evolution of host immunity. For example, the introduction of *Mycoplasma gallisepticum* into North America and its subsequent geographic spread created a gradient of bird populations that have had different lengths of time to evolve immunity. Comparisons of transcriptomic responses in house finches (*Haemorhous mexicanus*) from resistant versus naïve populations revealed that the former have constitutively higher expression of anti-inflammatory genes and Th1 response pathways (61). An alternative approach is to study host evolution following host-range expansion, leading to encounters with novel parasites. For instance, ancestrally marine threespine sticklebacks have repeatedly colonized freshwater lakes following Pleistocene glacial retreat. Once in freshwater habitats, sticklebacks encounter a variety of new parasites, including the cestode *S. solidus* (32).

Here, we present transcriptomic data to characterize the defense responses of sticklebacks that evolved in response to this novel parasite in a freshwater lake. We focus on the mechanisms that have allowed a general physiological response (fibrosis) to be co-opted for defense against a particular parasite (35). By comparing responses to a general stimulus (alum) and parasite-specific stimuli (cestode protein) across populations, we were able to highlight the roles of regulatory changes in facilitating the evolution of parasite resistance. Fibrosis is an ancient feature of metazoan life, essential for wound healing, and plays a role in parasite defense in various species. Most fish species have the capacity to initiate fibrosis in response to tissue damage (47). However, only some populations of threespine sticklebacks exhibit a fibrosis response to peritoneal tapeworm infections, suggesting that the fibrosis pathway has been co-opted to aid immunity to *S. solidus*. Our results reveal substantial co-option of ancient genetic pathways that remain intact, but whose expression level and timing are altered in freshwater populations that adapted to a cestode in the past 12,000 years. This finding adds to a growing list of studies documenting rapid evolutionary changes in immune gene expression in wild populations, enabling adaptation to changes in parasite communities (e.g (61–64). In sticklebacks, such adaptations lead to extensive population differences in transcriptome profiles (65, 66).

### General fibrotic responses to alum are mediated by change in T cell related genes with some patterns of self-regulation

When examining transcriptomic responses unique to alum, we observe strong signatures of complex changes in T cell activity. Like any tissue-level transcriptomic study, this could reflect either increased expression per cell or an increased abundance of a cell type (e.g., T cell proliferation or immigration) in the studied tissue. Similar changes are strongly associated with fibrotic phenotypes. Alum adjuvants are widely used because of their immunostimulatory functions and have been documented to have broad-scale effects on host T cell activity (67, 68). Specifically, alum adjuvants are known to affect antigen presentation and CD4^+^T cell activation, largely through direct interactions with dendritic cells (67, 68). This activation is typically thought to bias towards a Th2-type response (69). In contrast to these previous results, our study of stickleback responses to alum revealed broad patterns of changes in the expression of genes associated with a wide variety of T cell types, including CD4^+^ [i.e., *aimp1* and *lck* (70, 71)], CD8^+^ [i.e., *hmgb1* and *hmgb2a* (72, 73)], regulatory T cells [i.e., *nr4a1*, (74)], and even Th17 [i.e., *pdp2*, (75)]. We also failed to find strong evidence of a Th2 bias; for example, *anxa1a*, which induces a Th1 bias (76), is upregulated in response to alum in the stickleback. Our data indicate a more complex and nuanced interaction between alum and T cell activity in fish than in mammals, which is worth further investigation, especially considering the broad reactivity of the adjuvant across fish species (47).

Notably, these changes in T cell gene activity have significant overlap with gene expression modeling of fibrosis; changes in gene expression in response to alum are largely congruent with the effects on the fibrosis score (generally, ignoring treatment). This highlights a potential, previously undescribed role of T cell activity in mediating fibrotic responses to alum. While fibrotic changes and granulomas have been reported in response to alum in some animal models (77, 78), understanding of this link is limited. Additionally, ample evidence suggests a prominent role for T cell activity in fibrosis across tissue types (79, 80). The roles of T cells in regulating fibrosis are complex; Th1 cells are widely considered to be antifibrotic, whereas Th2 cells promote fibrosis (81). The complex patterns of T cell-associated gene expression captured in our dataset in response to alum are likely reflective of these multi-faceted roles of T cells in fibrosis. This association between T cell gene expression and both alum response and general fibrosis has potential implications for parasite response. Numerous studies have highlighted the importance of adaptive, and specifically T cell-related functions, in the response and resistance to parasites in sticklebacks (82–86). For example, reduced MHC diversity (which affects antigen presentation to T cells) is correlated with heightened parasite susceptibility (83). Other studies have suggested that *S. solidus* may modify T cell function during infections (84–86). The patterns described herein suggest that, at least in some populations, these important T cell changes may be linked to resistance phenotypes, such as fibrosis. Importantly, our data suggest that the associations between alum, T cell activity, and fibrosis are evolutionarily malleable. Notably, several alum-induced T cell genes had different associations with fibrosis depending on the fish population examined (**Figure 5B**).

Finally, while our bulk RNA-seq data suggest clear roles for T cell functions in response to alum, coarse deconvolution with existing head kidney cell type-specific markers suggests roles for multiple other immune cell types, specifically antigen-presenting cells, B cells, hematopoietic cells, and natural killer cells. Existing stickleback head kidney cell atlases have failed to identify T cell populations in sufficient numbers to determine markers, preventing any assessment of T cell proliferation using such approaches. Nevertheless, the proliferation of antigen-presenting cells and B cells, as observed in our data, could be directly linked to T cell function. Increased antigen-presenting cell populations likely support the activation of T cells (87, 88), while B cell proliferation is likely a consequence of increased T cell activity (89). Furthermore, we detected signatures of proliferation of both cell types in response to alum, which was associated with fibrosis, further suggesting that T cell activation and activity contribute to alum-induced fibrosis. Further validation of scRNA-seq markers will help disentangle the relative roles of changes in gene expression and cell type-specific proliferation in these processes.

### Defense responses to a novel parasite are heavily reliant on conserved, non-specific immune pathways

Although our results regarding alum are an interesting illustration of the dynamic nature of immune regulation over microevolutionary time, sticklebacks are not evolving to adapt to alum. Rather, they are evolving to defend against (or tolerate) diverse parasites, such as *S. solidus*. The differences (and similarities) in the responses of stickleback populations to *S. solidus* protein injection can provide more information about the process of adaptation to a novel parasite. In particular, to what extent do freshwater populations (which are frequently exposed to *S. solidus*) use deeply conserved immune regulatory pathways present in many vertebrates? Do they use pathways that are also present in ancestral marine fish that did not co-evolve with this cestode? Alternatively, are their responses significantly derived?

In general, cestode protein-induced changes were observed in a reduced set of genes compared to responses to alum, particularly when considering the main effects. However, cestode protein induced numerous unique changes in gene expression compared to alum, the majority of which were associated with largely conserved, non-specific immune responses. Specifically, unique cestode protein responses are most broadly characterized by changes in genes associated with inflammation and the activity of the highly conserved, multi-faceted immune transcription factor NF-κB. Specifically, we observed general trends of initial upregulation of NF-κB-related genes in response to cestode protein, which reverted to suppression by day 42 (**Figure 7**). NF-κB is a prominent immune regulator found across metazoans (90) that has frequently been co-opted into the defense response to parasites across taxa (91–94). Previous evidence suggests its role in the stickleback parasite response (65). The broad-scale activity of NF-κB, as well as numerous pathways for its activation, likely explains its central and repeated role in parasite defense (90).

Notably, reflecting the broad activity of NF-κB, we observed few indications of the induction of specific (i.e., adaptive) immune responses following cestode protein treatment. Instead, we observed general changes in inflammation-associated genes. Inflammatory responses are regulated by NF-κB activity (90) and are important components of general and innate host defense, including parasite defense (95). Numerous studies have highlighted the importance of respiratory bursts or ROS production, which are part of the inflammatory response, in the response of sticklebacks to *S. solidus* (96, 97). Thus, our results are consistent with prior findings and further emphasize the role of inflammatory processes in the host response to parasites. Furthermore, inflammation is often positively associated with or even triggers fibrosis (98), potentially explaining the population-specific expression patterns observed. Notably, we observed tighter regulation of inflammation processes in response to cestode protein in GOS fish, mostly through increased activation of anti-inflammatory genes, including *dusp10* (99), *tax1bp1a* (100), *apc* (101), and *mef2cb* (102). The suppression of inflammatory responses following cestode protein exposure in GOS fish likely mechanistically contributes to reduced fibrotic responses.

### Further evidence supporting the role of candidate fibrosis gene, *spi1b*, in cestode defense

Finally, while general, ancestral defense responses were most common in characterizing the response to cestode protein, we also observed a response that is specific to sticklebacks’ defense against cestode protein: the induction of a key candidate fibrosis gene, *spi1b*. *Spi1b* encodes a transcription factor that plays a role in fibrosis responses (58). In sticklebacks, mounting genomic evidence suggests that evolutionary divergence in this gene and/or surrounding regulatory regions underlies the differences in the observed fibrosis phenotypes across populations (35). CRISPR/cas9 editing of *spi1b* alters fibrosis phenotypes in sticklebacks, and pharmacological inhibition of the *spi1b* protein product (PU.1) leads to suppressed fibrosis response (40). Furthermore, independent studies in other stickleback populations have documented the activation of *spi1b* in response to *S. solidus* (103). Our data demonstrate that *spi1b* activation in response to cestode proteins is tightly regulated. *Spi1b* was rapidly activated following injection of cestode protein, but by the end of the experiment, it was suppressed in treated fish, further indicating the importance of regulating potentially harmful defense responses. Finally, *spi1b* expression was positively associated with fibrosis in two of the three populations (see below for further discussion of population differences). This positive association is consistent with an experiment showing that drug inhibition of *spi1b* (by db1976) suppressed the fibrosis response in lab-raised sticklebacks (40).

### Comparison of responses between treatments reveals the importance of response reregulation in effective parasite defense

As noted previously, significant differences were observed in the magnitude of responses to alum and cestode proteins, as well as the distribution of differentially expressed genes across model terms of interest. Specifically, we observed a much stronger response to alum, with many more genes significantly differentially expressed as a result of the main treatment effect. In comparison, cestode protein induced considerably weaker changes in gene expression. However, responses to cestode protein were also notable in that they exhibited much stronger signatures of changes in gene expression over time (treatment × time interaction). Genes significant for a treatment × time interaction were nearly five times more abundant than main effect genes in response to cestode protein; in contrast, alum main effects were seven times more abundant than treatment × time interactions. These patterns are reflected when we consider the direct overlap in shared gene responses between the two responses. While direct overlap among genes significant for main effects across treatments was limited (13 genes total; only four immune), we observed significant overlap in genes persistently responsive to alum (significant main effect of treatment) and those that responded transiently to cestode protein producing a treatment × time interaction (54 genes, eight immune). These genes serve diverse immune roles but were unified in their broad patterns; all but one were consistently upregulated in response to alum, and were also upregulated in response to cestode protein on day 1, but were then downregulated by day 42.

These patterns of gene-specific regulation were observed even when considering unique alum and cestode protein-responsive genes. For example, nine of our alum-responsive T cell-associated genes were significantly dependent on the interaction of alum × time, starting out activated in alum-treated fish, but reaching a suppressed state by the end of the experiment. Similarly, inflammatory responses to cestode proteins are tightly regulated over time in some populations. When considering broader scale trajectories using DPAC, we observed some indication of similar patterns, specifically potential regulatory changes or negative feedback between days 42 and 90 in RSL only (fast fibrosis; cestode protein fibrosis), though our 90 day sampling point entailed smaller sample sizes. However, patterns of gene expression suggest that, at a minimum, the regulation of targeted genes and consequently fibrosis is essential. Longer-scale experiments (and with higher temporal resolution) will be key in exploring these regulatory pathways and their roles in fibrosis recovery.

Combined, these results provide evidence that the regulation of defense responses to novel parasites such as *S. solidus* is likely a key component of immune adaptation. This is particularly true in the case of fibrosis, which can have significant fitness consequences for the host (35, 39, 104). Costly defense responses are theorized to be tightly regulated by negative feedback loops to mitigate potential immunopathologies (17, 105, 106). Here, we provide empirical evidence to support these theories; our data suggest that the co-option of fibrosis to defend against novel parasites co-evolved with the associated increased regulation of the phenotype.

### Shared and divergent parasite responses of stickleback populations

Phenotypic measures of fibrosis responses were highly variable across populations (36; Choi et al., in prep)^1^. RSL fish exhibited a faster fibrosis response to alum than GOS and SAY but were also able to recover (reduced fibrosis by day 90), whereas GOS and SAY retained high fibrosis. RSL fish also responded to cestode protein, whereas GOS and SAY fish did not. These striking phenotypic differences between populations were not reflected in the transcriptomic data presented here. Our general models detected only modest population-level differences in gene expression responses to alum and cestode proteins. These patterns suggest that differences in a small subset of genes may have a strong impact on the observed phenotypic patterns. Alternatively, phenotypic patterns may be better explained by differences in post-translational regulation or localized peritoneal changes, which are difficult to measure due to low tissue yield (especially in fibrotic fish). However, our comparative analyses of gene expression data combining multiple population-independent and population-specific models suggest that population divergence in a limited number of fibrosis-related genes may strongly impact phenotypic differences.

Most notably, a comparison of the results from our treatment- and fibrosis-focused models sheds light on a handful of genes that are divergent across populations and may explain the strong variation in treatment response. Specifically, several genes that responded to injection were also related to fibrosis score, including genes with both a main effect of alum and cestode protein × time interactions, T cell-associated genes, and *spi1b*. Several of these genes were significantly differentially associated with fibrosis across populations (*cyldl, irf4a, klf2b, mrc1b, prkd2, rbx1*, sp*i1b*, and *tnfaip8l2b*), nearly all of which displayed similar patterns. Specifically, most of these genes were tightly associated with fibrosis scores in GOS and SAY fish (which displayed slower fibrosis phenotypes, and only responded to alum), but not in RSL (fast fibrosis response to both alum and cestode protein). Furthermore, RSL fish frequently had the highest levels of expression of all fibrosis genes (those significant for both main and interactive effects). The decoupling of gene expression-fibrosis patterns and higher general expression of fibrosis genes in RSL suggests that constitutive upregulation (i.e., constantly “on”) of these genes contributes to the patterns of faster fibrosis induction and unique fibrotic responses to cestode protein in RSL (36). By constitutively expressing these genes, resistant fish may be able to immediately trigger fibrosis responses to parasites, in contrast to tolerant/ancestral fish, which likely first must undergo steps to increase the transcription of these genes before inducing fibrosis phenotypes (hence explaining the observed strong positive association between these genes and fibrosis phenotypes).

These findings build upon prior results, which suggested that constitutively higher expression of immune genes may contribute to parasite resistance in other stickleback populations (49). Previous studies have also identified constitutive differences between resistant and tolerant/ancestral populations in standing levels of neutrophils; resistant populations had constitutively more neutrophils than others, which was hypothesized to support fast parasite responses (37). Our discovery that neutrophil marker genes are significantly and positively associated with fibrosis strongly supports this hypothesis. Recent work has also shown that some stickleback populations are severely fibrotic, even in the absence of an immune challenge, and evolution has apparently driven them to an anticipatory response that is always on (Choi et al., in prep). Finally, a shift toward constitutive activation of these genes likely contributes to the observed patterns of immune regulation; constitutive expression of genes that can induce costly phenotypes likely results in selective pressure for the observed patterns of enhanced regulation.

Our population comparative models further highlight potentially important population-level divergences in responses. While population-specific model responses to both alum and cestode proteins were largely congruent when comparing our ancestral population (SAY) to either freshwater population (GOS or RSL), we noted a significant divergence in response to cestode protein between our two freshwater populations, GOS and RSL (*r* = −0.217, *p* = 0.0118). This means that genes that tend to be upregulated in response to cestodes in GOS tend to be downregulated in the other, a remarkable reversal for populations that have diverged for only approximately 12,000 years. These patterns are potentially evidence of the divergent evolution of parasite response strategies (35), although recent adaptive introgression into GOS may have dampened our signature (40).

Finally, it should be noted that our study is limited by replication within population types (i.e., only one resistant/tolerant/ancestral population). However, these phenotypic patterns have been observed repeatedly across numerous stickleback populations (31, 34, 35, 39), and our gene expression patterns show similarities with prior studies involving other populations with similar phenotypes (49, 65, 107, Choi et al., in prep). In contrast, a recent multi-species investigation of the transcriptomic patterns of fibrosis suggests a strong divergence in the underlying mechanisms (107). Thus, future studies expanding across multiple populations of sticklebacks with varying degrees of parasite resistance are necessary to fully interrogate the repeatability and generalizability of the findings reported here.

The above results show that the rapid evolution (over a few thousand years) of population differences in immune phenotypes can be achieved with relatively few differentially expressed genes and shifts in the timing of up- and downregulation. This insight is consistent with transcriptomic studies of recently evolved immunity in other sticklebacks (e.g (66, 108) and in other organisms. For example, house finches in North America have adapted to the invasive bacterial pathogen *Mycoplasma*. Gene expression has diverged between finch populations from Virginia (where infection is long established) and Arizona (where infection is recent) (61). However, only a few genes (and only three immune genes) exhibited significant interactions between the finch population and pathogen exposure treatment, similar to our short list of treatment × population interactions. Similarly, a nematode parasite induced a modest number of differentially expressed genes in two species of eels (one native host and one invaded host) (64). Populations of freshwater snails in New Zealand exhibit divergent gene expression responses to trematode parasites (109), as do populations of *Biomphalaria* snails in response to allopatric versus sympatric *Schistosoma* genotypes (110). Collectively, these and other studies present a growing body of research showing that relatively modest changes in gene regulation can play a major role in the rapid evolution of immunity (111).

### Conclusions

Our transcriptomic analyses highlight the gene expression responses underlying divergent host responses to a novel parasite, with a particular focus on potent defense responses (fibrosis). Our results provide new insights into both the general mechanisms of fibrosis (i.e., T cell activity) and potential parasite-specific responses (i.e., NF-κB and inflammation). Most importantly, we leveraged comparative analyses to highlight the importance of immune regulation in fibrosis and parasite responses. These findings highlight the importance of the evolution of immune regulatory mechanisms as a step towards the co-option of fibrosis for parasite defense. Additionally, we provide new evidence that the evolution of elevated constitutive expression of fibrosis-associated genes may contribute to the rapid induction of fibrosis associated with enhanced parasite resistance. Taken together, our results suggest that the evolution of costly parasite defenses involves changes in the baseline expression of key genes and the evolution of associated immune regulatory mechanisms to prevent self-harm associated with this effective but costly immune defense. These results provide important empirical evidence to support a robust theoretical study of the evolution of defense response optima.

## Data Availability

The datasets presented in this study can be found in online repositories. The names of the repository/repositories and accession number(s) can be found below: https://github.com/lfuess/InjectionMS, GitHub.

## Footnotes

1 Choi ES, Flanagan BA, Bolnick DI, Berini J, Hund AK, Albright A, et al. Heritable variation in fibrosis response among lake populations of threespine stickleback. in prep.

## Supplementary material

Supplementary Table 1 Table of sample sizes for each population-treatment-time point combination.

Supplementary Table 2 Summary of results of test of equal proportions considering overlap of all pairwise combinations of effects of interest across our alum and cestode protein specific models.

Supplementary Table 3 Summary of fibrosis model results for selected genes of interest. Table displays p values for each main model term (fibrosis, population, and interaction) and results of post-hoc testing for differences in expression across populations. Genes shown are those significant for fibrosis or fibrosis x population interaction which were also significantly differentially expressed for a model term of interest in our alum and/or cestode protein models.

Supplementary Table 4 Summary of population-specific fibrosis models for genes of interest. Shown are p values for the population x fibrosis interaction in the global (all populations) model, and then p values and coefficients for the fibrosis term in population-specific GLMs. Genes shown are those which were significant for the population x fibrosis interaction in the global model, and were also significantly differentially expressed for a model term of interest in our alum and/or cestode protein models.

Supplementary File 1 Summary of sensitivity testing results. Displayed are differential expression results for model runs using the top 50% and top 75% most expressed genes. Each sheet is a specific model (alum, CP, fibrosis), with a final sheet summarizing results from alum-CP overlap comparisons for each run. Highlighted rows indicate comparisons where results differ between model runs (50% vs. 75% cutoff).

Supplementary File 2 Summary of analysis of differential expression of identified putative immune genes in the lower 50% expressed genes. Included is a list of keywords used to search Gene Ontology terms and identify putative immune genes. Additional tabs report overrepresentation and overlap (between alum and CP models) statistical analysis. Finally, individual tabs list differentially expressed genes and relevant statistics for each model (alum, CP, fibrosis).

Supplementary File 3 Summary of significantly differentially expressed genes from each of the three main models (alum, CP, fibrosis). Reported are gene names, annotations (where possible), and coefficient and p values for each relevant model term (excluding population, time, and population x time interactions).

Supplementary File 4 Summary of significantly differentially expressed genes from each of the population specific treatment (alum and CP) models. Reported are gene names, annotations (where possible), and coefficient and p values for treatment and treatment x time interaction terms. Sheets are labeled based on population and treatment.

Supplementary Figure 1 Schematic displaying experimental design setup for data collection; fish from three populations: Gosling (GOS), Sayward (SAY), and Roselle (ROS) were exposed to one of three treatments: phosphate buffered saline control (PBS), alum adjuvant (alum), or cestode protein (CP). Fish were then sampled at Days 1, 10, 42, and 90 post injection. Fibrosis was scored and head kidneys were removed for 3’ TagSeq transcriptomics.

Supplementary Figure 2 Scatterplots displaying relationship between responses to **(a)** treatment main effects, **(b)** treatment by time effects and **(c, d)** treatment by population effects across alum and cestode protein models. Graphs show all genes which were tested in both models. Points are colored based on the relationship of the coefficients for each model, wherein blue dots indicate congruence across alum and cestode protein effects and red points indicate divergence. Pearson correlation results are displayed for each comparison.

Supplementary Figure 3 Line plots displaying proportional normalized read count values of genes of interest in treatment and control groups over time. Plots are paired with alum response on the left (indicated with star icon) and cestode protein response on the right (indicated with worm icon). Dotted lines indicate control values whereas solid lines indicate treatment values. As there were no significant population effects lines are shown for all fish within a treatment combined across populations.

Supplementary Figure 4 Line plots displaying proportional normalized read count values of uniquely alum-responsive T cell genes which were differentially expressed in response to alum over time and across populations. Plots show trajectories of treatment and control groups over time. Lines are colored based on populations. Dotted lines indicate control values whereas solid lines indicate cestode protein values.

Supplementary Figure 5 Violin plot with inlaid box and whisker plot for putative immune genes which displayed opposing responses to cestode protein in the population specific modules. * Indicates significance of the treatment term in the model. Plots are paired with GOS on the left and RSL on the right, indicated by the small colored letter in the upper righthand corner of each plot.

Supplementary Figure 6 Trajectory plot of response to **(a)** alum and **(b)** cestode protein for each population independently based on genes which were significant for treatment or any interaction term including treatment across both alum and CP (2230 total). Closed circles indicate centroids for treated fish at each time point, whereas open circle indicates centroid for control fish. Arrows indicate trajectory between time points. Crosses indicate relative spread of data points at each time point. Axes are not standardized across plots for simplified visualization.
