# Supplementary figures and images for: Changes in gene regulation are associated with the evolution of resistance to a novel parasite
Source: Front Immunol. 2026 Feb 26;17:1697157. doi: 10.3389/fimmu.2026.1697157 (PMC12979160; doi:10.3389/fimmu.2026.1697157)

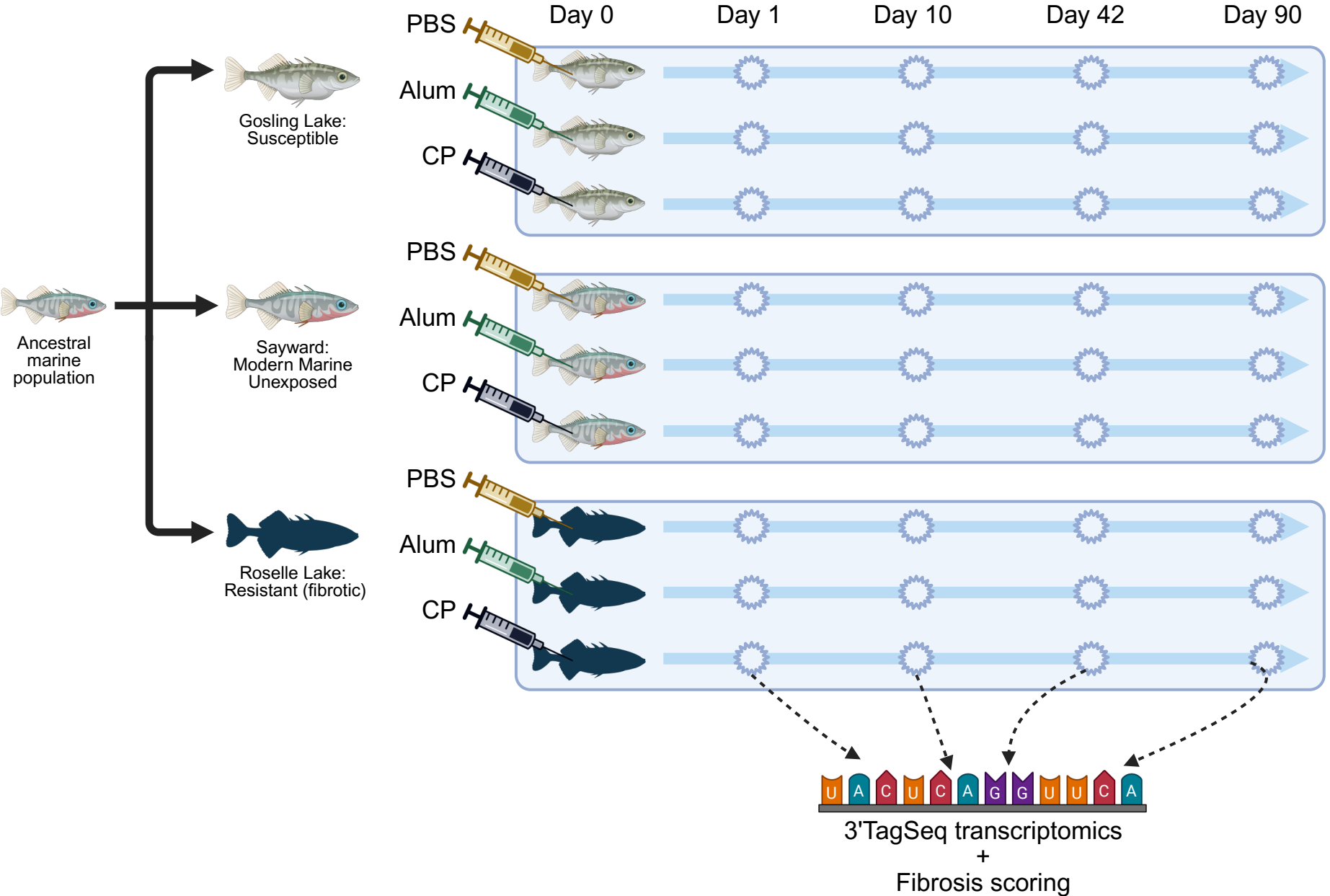

Supplement: Supplementary Figure 1 — Schematic displaying experimental design setup for data collection; fish from three populations: Gosling (GOS), Sayward (SAY), and Roselle (ROS) were exposed to one of three treatments: phosphate buffered saline control (PBS), alum adjuvant (alum), or cestode protein (CP). Fish were then sampled at Days 1, 10, 42, and 90 post injection. Fibrosis was scored and head kidneys were removed for 3’ TagSeq transcriptomics. [file Image1.pdf]

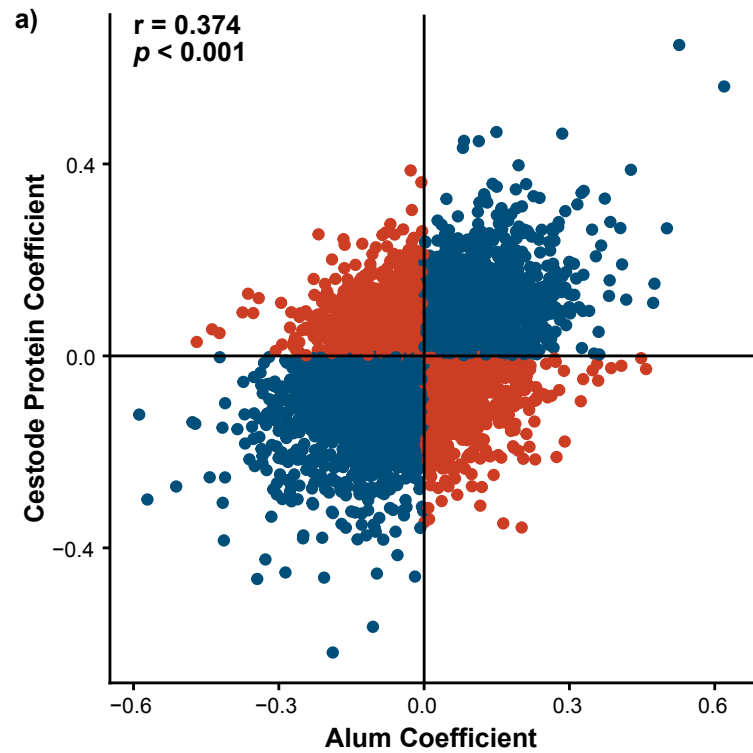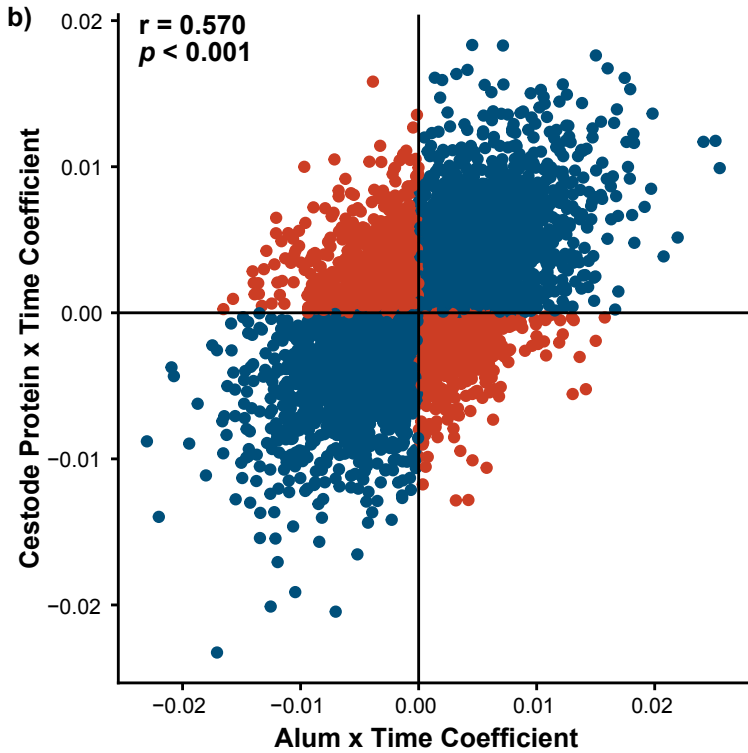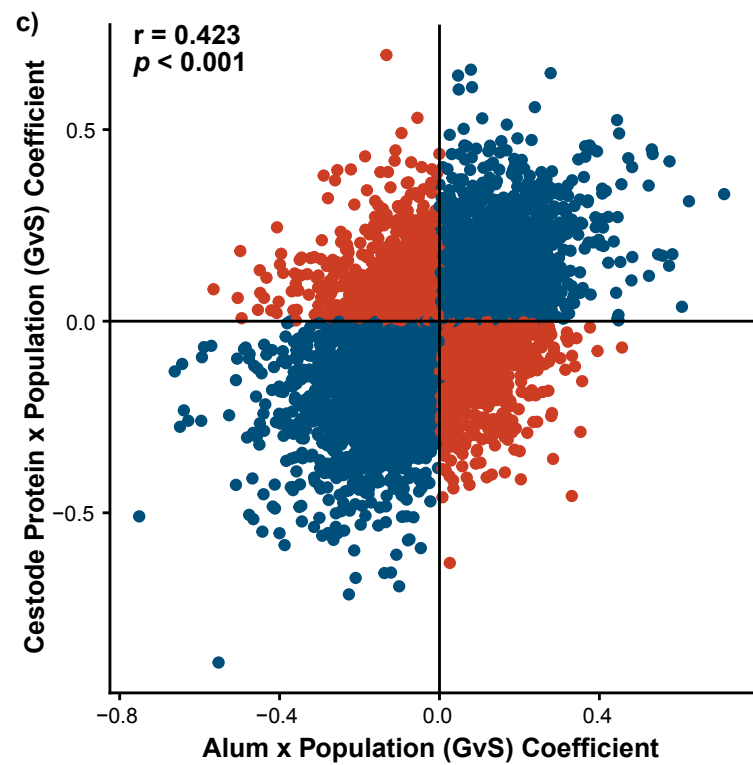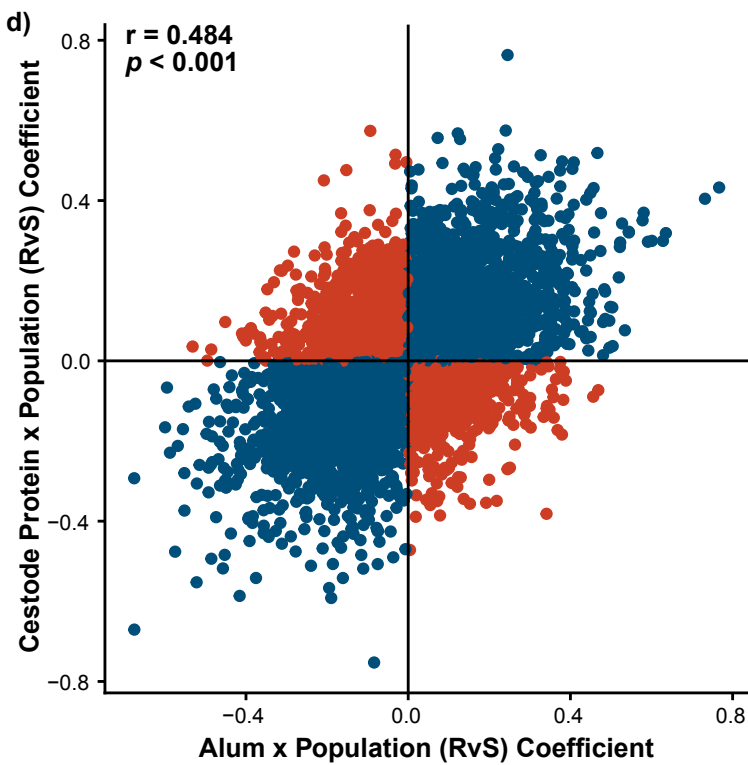

Supplement: Supplementary Figure 2 — Scatterplots displaying relationship between responses to (a) treatment main effects, (b) treatment by time effects and (c, d) treatment by population effects across alum and cestode protein models. Graphs show all genes which were tested in both models. Points are colored based on the relationship of the coefficients for each model, wherein blue dots indicate congruence across alum and cestode protein effects and red points indicate divergence. Pearson correlation results are displayed for each comparison. [file Image2.pdf]

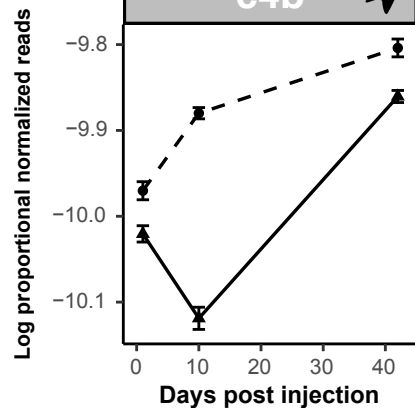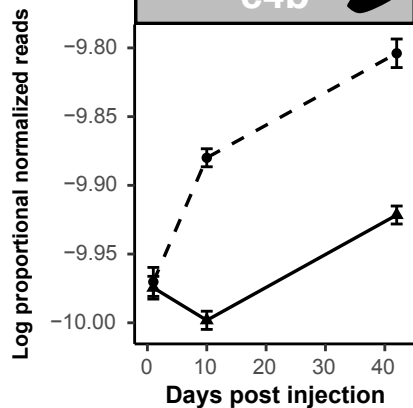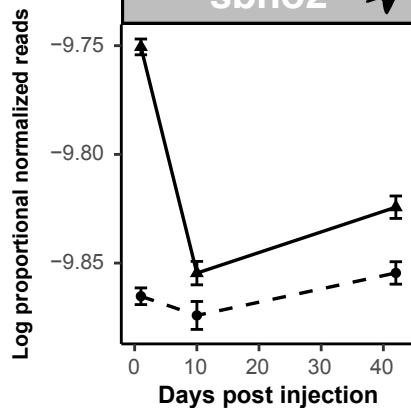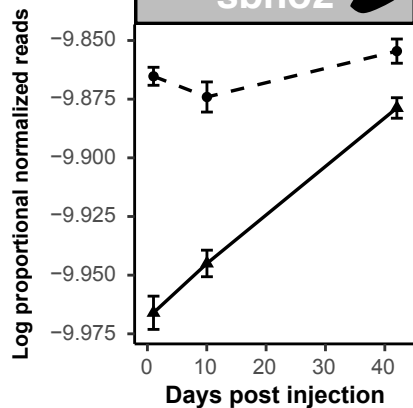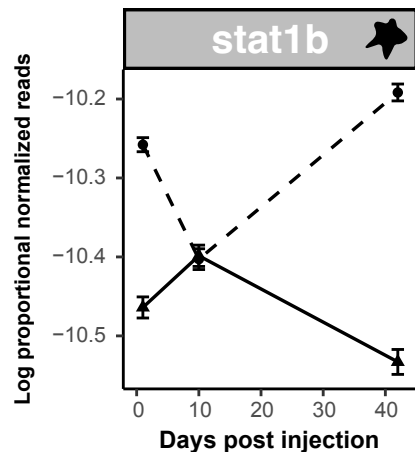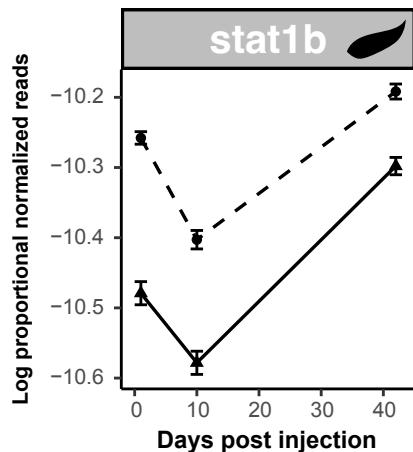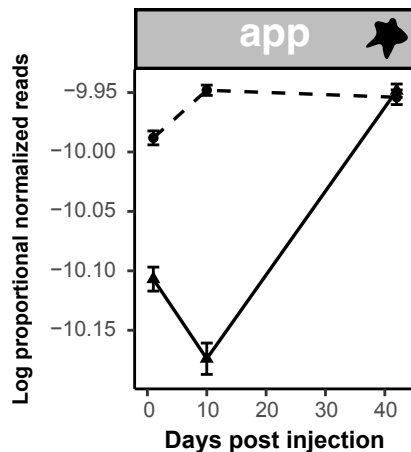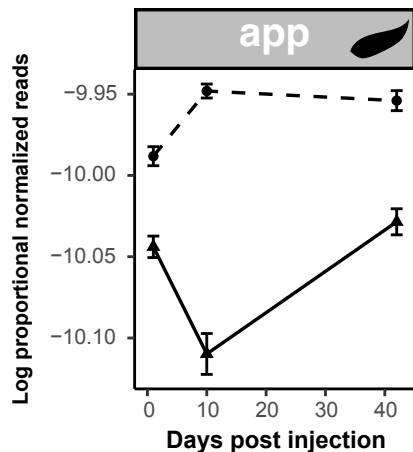

Supplement: Supplementary Figure 3 — Line plots displaying proportional normalized read count values of genes of interest in treatment and control groups over time. Plots are paired with alum response on the left (indicated with star icon) and cestode protein response on the right (indicated with worm icon). Dotted lines indicate control values whereas solid lines indicate treatment values. As there were no significant population effects lines are shown for all fish within a treatment combined across populations. [file Image3.pdf]

Treatment    ●-- PBS    ▲-- Alum    Population    ●-- SAY    ●-- GOS    ●-- RSL

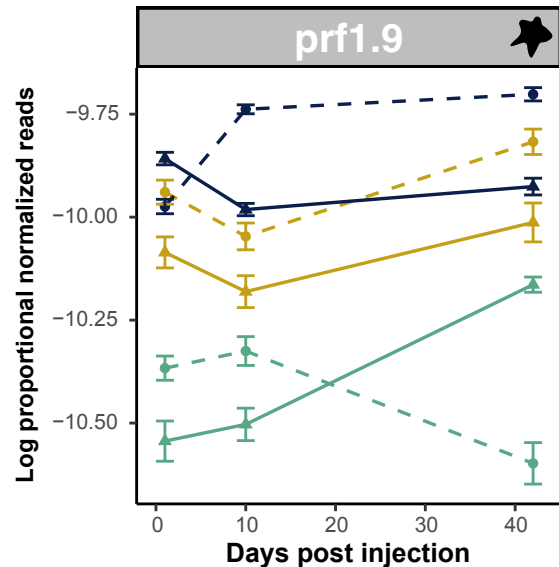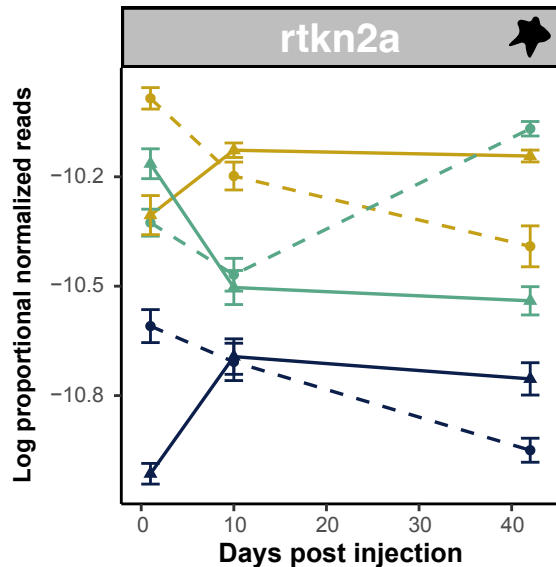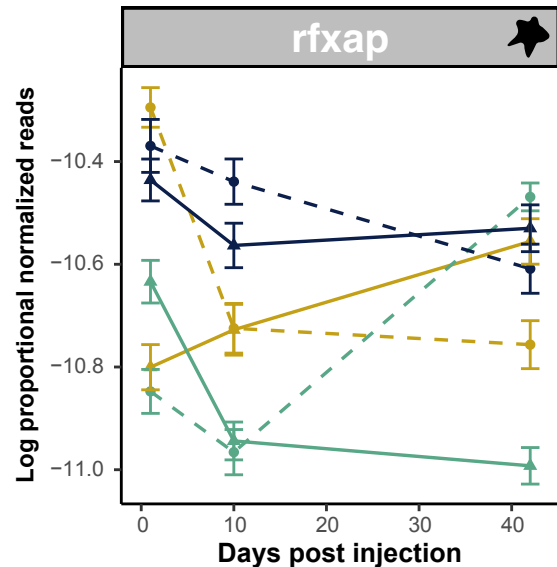

Supplement: Supplementary Figure 4 — Line plots displaying proportional normalized read count values of uniquely alum-responsive T cell genes which were differentially expressed in response to alum over time and across populations. Plots show trajectories of treatment and control groups over time. Lines are colored based on populations. Dotted lines indicate control values whereas solid lines indicate cestode protein values. [file Image4.pdf]

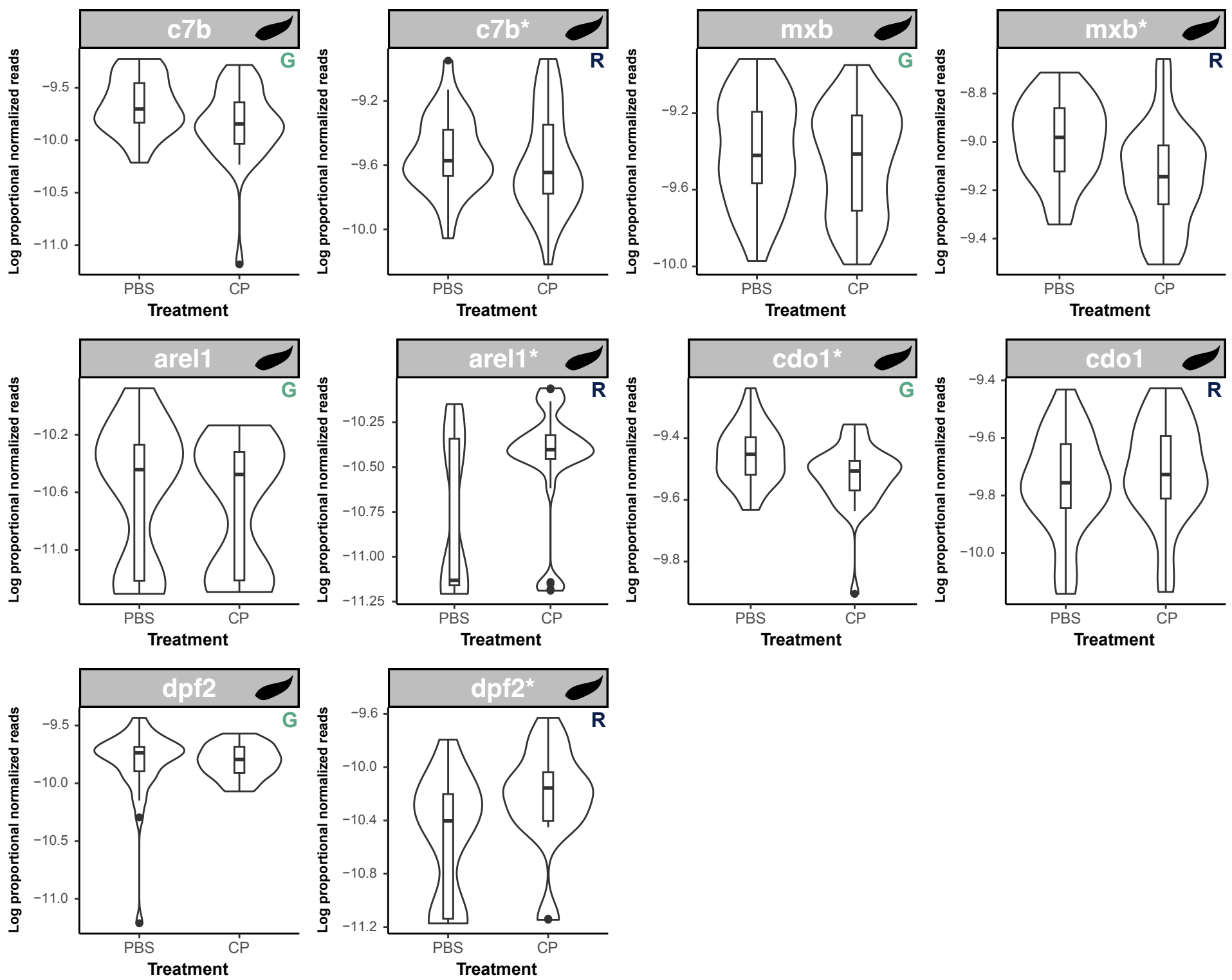

Supplement: Supplementary Figure 5 — Violin plot with inlaid box and whisker plot for putative immune genes which displayed opposing responses to cestode protein in the population specific modules. * Indicates significance of the treatment term in the model. Plots are paired with GOS on the left and RSL on the right, indicated by the small colored letter in the upper righthand corner of each plot. [file Image5.pdf]

a)

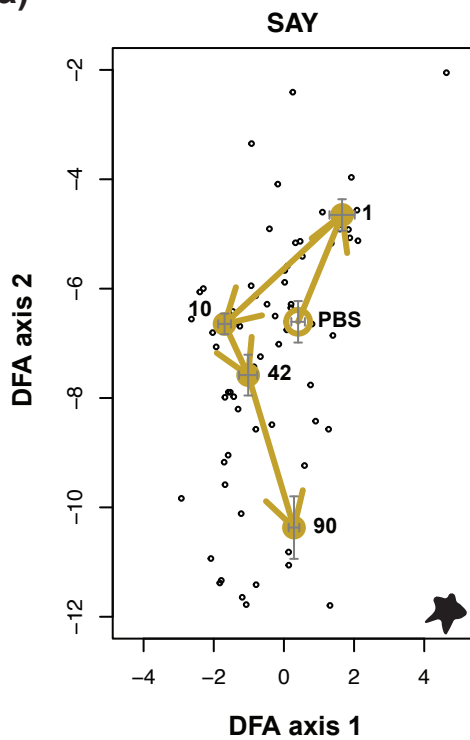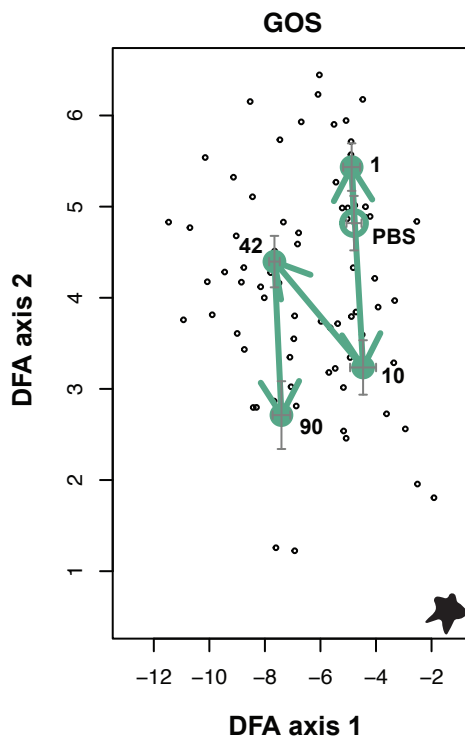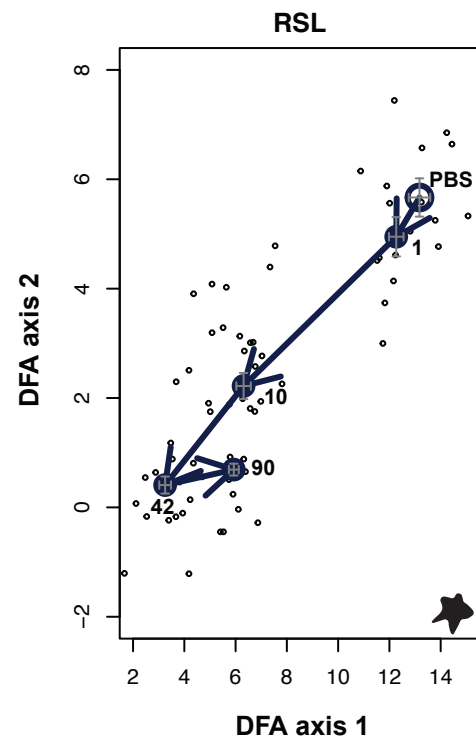

b)

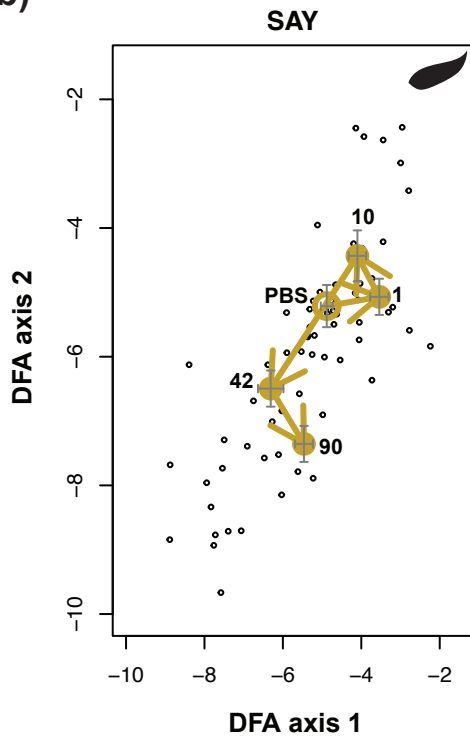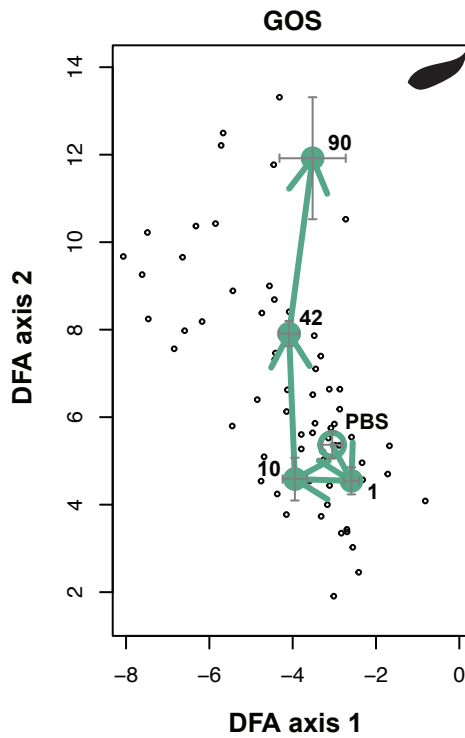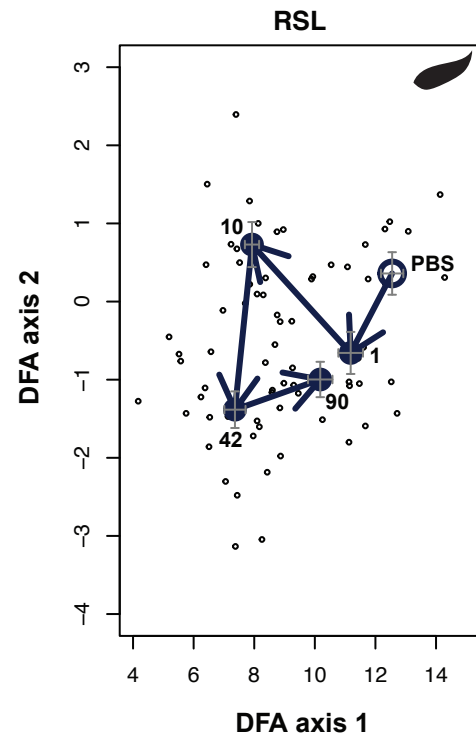

Supplement: Supplementary Figure 6 — Trajectory plot of response to (a) alum and (b) cestode protein for each population independently based on genes which were significant for treatment or any interaction term including treatment across both alum and CP (2230 total). Closed circles indicate centroids for treated fish at each time point, whereas open circle indicates centroid for control fish. Arrows indicate trajectory between time points. Crosses indicate relative spread of data points at each time point. Axes are not standardized across plots for simplified visualization. [file Image6.pdf]
